# Supplementary material for: Lost in Translation: An OSCE-Based Workshop for Helping Learners Navigate a Limited English Proficiency Patient Encounter
Source: MedEdPORTAL. 2021 Mar 17;17:11118. doi: 10.15766/mep_2374-8265.11118 (PMC7970641; doi:10.15766/mep_2374-8265.11118)
Supplement: Supplementary file 1 — Description of Workshop Components.docxChecklist.docxPreworkshop OSCE.docxPanel Discussion.docxWorking With Health Care Interpreters.pptxMap of Postworkshop OSCE.docxFacilitator Guide for Interactive Q&A.docxDebriefing.docxPostworkshop OSCE.docx [file mep_2374-8265.11118-s001.zip › A. Description of Workshop Components.docx]

**Appendix A**: Description of Workshop Components: Terminology, Activities, Time Duration, and Participants

| Term | Description | Time Duration | Activity | Number of participants |
| --- | --- | --- | --- | --- |
| Lost in Translation | The name of our curriculum, which includes the workshop and 2 OSCE’s | 5 hours spread over 4 weeks | Combination of observing, listening, interactive question and answer session, and hands on activity | Residents (n=40)  Group 1: 10 were OSCE participants and attended the workshop  Group 2: 30 other residents who only attended the workshop |
| Pre-Workshop OSCE | Jaundice OSCE, which took place 1 month prior to the workshop | Each case was 10 minutes and 2 OSCE rooms ran simultaneously for a total of 2 hours | Participating in the OSCE | Group 1 (n=10) |
| Workshop | Consisted of:  a panel discussion,  a presentation on best practices, watching a video demonstration, observing OSCE’s, and the interactive question and answer session  The interactive question and answer session took place simultaneously as the scenarios, but in a separate room | 3 hours (completed in 1 afternoon)  Panel Discussion: 30 minutes  Presentation: 30 minutes  Video demonstration: 10 minutes  Observing scenarios: 2 hours  Question and answer session: 30 minutes | Combination of observing, listening, interactive question and answers, and hands on activity | Both groups attended the workshop  (n=10) |
| Post-Workshop OSCE | Abscess OSCE, which takes after the workshop | Each case was 10 minutes. We had 3 simultaneous rooms for the OSCE, for a total of 2 hours | OSCE performed by the same 10 residents who completed the pre-workshop OSCE; these were observed by an audience of residents | Group 1 (n=10) |
