# Supplementary material for: Lost in Translation: An OSCE-Based Workshop for Helping Learners Navigate a Limited English Proficiency Patient Encounter
Source: MedEdPORTAL. 2021 Mar 17;17:11118. doi: 10.15766/mep_2374-8265.11118 (PMC7970641; doi:10.15766/mep_2374-8265.11118)
Supplement: Supplementary file 1 — Description of Workshop Components.docxChecklist.docxPreworkshop OSCE.docxPanel Discussion.docxWorking With Health Care Interpreters.pptxMap of Postworkshop OSCE.docxFacilitator Guide for Interactive Q&A.docxDebriefing.docxPostworkshop OSCE.docx [file mep_2374-8265.11118-s001.zip › B. Checklist.docx]

**Appendix B: Checklist**

**Learner Name:**

**Case:**

**Time:**

**Instructions for observer:**

- Only check the items that you observe the learner perform during the OSCE; at the end, add up all the items that were performed to create a score out of 16.
- Record the time in minutes and seconds at which the learner identifies the need for interpreter (e.g., “I need an interpreter”).
- Document observations you noticed from the encounter (e.g., the learner refers to the interpreter as a “translator”, the learner spoke fast and in long run on sentences, etc.) in the “Notes” section.

**The learner should:**

- Introduce him/herself and explain his/her role
- Identify the need for interpreter services within 90 seconds (e.g., I need an interpreter)
- Explain to the caregiver the reason for why an interpreter is needed
- Place iPad/phone in appropriate location when using electronic interpreter OR positions him/herself appropriately to work with the in-person interpreter
- Briefly explain the purpose of the interview to the interpreter, especially for sensitive appointments (such as suspected child abuse)
- Ask the patient one question at a time
- Present information at a pace that is easy to follow for both patient and interpreter; that is, give information in “digestible chunks” and/or allow appropriate pauses
- Avoid using medical jargon and/or acronyms
- Maintain direct eye contact with the patient instead of with the interpreter
- Ask questions in the first person (e.g. “Do you feel…” versus “Does mom feel…”)
- Utilize teach back to ensure patient comprehension (e.g., “Can you explain what you understand of the plan to me?”)
- Lean on interpreter for cultural cues (e.g., patient does not want to be touched, no eye contact is normal, male speaking on behalf family/wife is normal, etc.)
- Nonverbal body communication should be reassuring (e.g., mannerisms, facial expressions, body language)
- Talk at an appropriate volume (e.g., does not talk louder due to working with an interpreter)
- Address the issues that were of concern to the patient
- Acknowledge and respond to the beliefs, concerns, and expectations about the patient’s problems (e.g., if parent is concerned about the evil eye affecting the patient)

**Notes**:
