# Supplementary material for: Lost in Translation: An OSCE-Based Workshop for Helping Learners Navigate a Limited English Proficiency Patient Encounter
Source: MedEdPORTAL. 2021 Mar 17;17:11118. doi: 10.15766/mep_2374-8265.11118 (PMC7970641; doi:10.15766/mep_2374-8265.11118)
Supplement: Supplementary file 1 — Description of Workshop Components.docxChecklist.docxPreworkshop OSCE.docxPanel Discussion.docxWorking With Health Care Interpreters.pptxMap of Postworkshop OSCE.docxFacilitator Guide for Interactive Q&A.docxDebriefing.docxPostworkshop OSCE.docx [file mep_2374-8265.11118-s001.zip › C. Preworkshop OSCE.docx]

| **Appendix C: Pre-workshop OSCE**  **SIMULATION CASE TITLE: Making Students Aware of Language Services for Patient Encounter: Jaundice**  **AUTHORS: Jan Fune, MD**  **LEARNER AUDIENCE: Pediatricians, pediatric residents, and medical students** | |
| --- | --- |
| **PATIENT NAME: Jorge Lopez**  **PATIENT AGE: 4 days old**  **CHIEF COMPLAINT: Breastfeeding Jaundice**  **PHYSICAL SETTING: Inpatient unit** | |
|  | |
| **Brief narrative description of case** | Patient is a 4-day old newborn male directly admitted from his pediatrician’s office to the hospital for breasting jaundice. When the learner does the admission, the nurse notifies the learner that the mom does not understand why she was admitted to the hospital, and that she keeps taking her baby out of the phototherapy lights. Mom is Spanish speaking and is accompanied by her friend.  The overall learner goals are to (1) identify the need for using an interpreter and (2) convey the importance of not using laypeople as ad hoc interpreters, and (3) work with the interpreter in accordance to the guidelines presented in the workshop. |
| **Primary Learning Objectives** | - Recognize the need for language services - Explain need for language services to patient/caregiver(s) - Demonstrate consistent eye contact with patient/caregiver(s) - Exemplify speaking in digestible phrases |
| **Critical Actions** | The learner should:   - Introduce him/herself and explain his/her role - Identify the need for interpreter services within 90 seconds - Explain the reason for why an interpreter is needed - Place iPad/phone in appropriate location OR positions him/herself appropriately to work with the in-person interpreter - Briefly explain the purpose of the interview to the interpreter, especially for sensitive appointments - Ask the patient one question at a time - Present information at a pace that is easy to follow for both patient and interpreter; that is, give information in “digestible chunks” and/or allows appropriate pauses - Avoid using medical jargon and/or acronyms - Maintain direct eye contact with the patient instead of with the interpreter - Ask questions in the first person; e.g. “Do you feel…” - Utilize teach back to ensure patient comprehension - Lean on interpreter for cultural cues (e.g., patient does not want to be touched, no eye contact is normal, male speaking on behalf is normal, etc.) - Nonverbal body communication should be reassuring; e.g., mannerisms, facial expressions, body language - Talks at an appropriate volume (e.g., does not talk louder due to working with an interpreter) - Address the issues that were of concern to the patient - Acknowledge and respond to the beliefs, concerns, and expectations about the patient’s problems |
| **Learner Preparation or Prework** | Patient is a 4-day old newborn male directly admitted from his pediatrician’s office to the hospital for jaundice related to breastfeeding. When you go to do the admission, the nurse notifies you that the mom does not understand why she was admitted to the hospital, and that she keeps taking her baby out of the phototherapy lights. |

| **Initial Presentation** | | | |
| --- | --- | --- | --- |
| **Initial vital signs** | BP 90/60, HR 140, RR 40, Temp 98.3F, Weight: 3.5 kg | | |
| **Overall Setting and Appearance** | The setting takes place in the inpatient unit. Mom is holding her baby in her arms. The baby is well appearing, not fussy, but is jaundiced. | | |
| **Confederates (e.g., standardized participants) and their roles in the room at case start** | Patient’s mom and mom’s friend are present with the patient.  **Instructions for the parent:**   - Do not speak in English - Inform the learner that you do not understand why your baby was admitted - Please appear anxious and ask several questions - Sample questions: What is jaundice? How did my baby get jaundice? Why do we need to be admitted? What is the treatment for this? - If the learner offers an interpreter, please insist that your friend can interpret for you - Only accept the interpreter once the learner has stated why a certified one is needed - If the resident uses medical jargon, tell them that you do not understand - Optional: may add cultural competency piece to encounter, such as concern for evil eye   **Instructions for the parent’s friend:**   - Do not introduce yourself unless the learner asks to identify you - Remember not to act as if you are a professional or certified interpreter - Interpret only segments of the dialogue - Do not do exact translations of mom’s dialogue - Add your own questions, comments, or dialogue apart from mom’s   **Instruction for interpreter:**   - Only enter the encounter if the learner requests an interpreter - Introduce self to learner and parent - Do not ask the learner for a description of the encounter or reason for interpreter request (learner should provide this information to you unprompted) - May interpret consecutively or simultaneously depending on how the case unfolds   **Instruction for observer**   - Position yourself in such a way that you can clearly see all the participants’ faces in the room   **Parent’s opening statement**: “His pediatrician said we had to be admitted to the hospital, but I do not understand why” | | |
| **HPI** | **Volunteered:**   - This is mom’s first baby - He was discharged on day of life 2 and was doing well at home - Mom went to pediatrician today for routine visit and was told that the baby needed to be admitted for jaundice   **Must be asked:**   - Pregnancy related questions – Baby was born full term, vaginally, with no complications during pregnancy or delivery - Mom’s past medical history – History of eczema - Formula or breastfed? – Baby is exclusively breastfeeding - Supply? – Mom believes that her milk supply is in - Frequency of feeds? – About every 2 – 3 hours - How are feeds going? – Baby latches well and has a strong, vigorous suck - How is baby acting? – Normal, mom has no concerns | | |
| **Past Medical/Surgical History** | **Medications** | **Allergies** | **Family History** |
| None | None | None | Not pertinent |
| **Physical Examination** | | | |
| **General** | Well appearing, adequately hydrated, facial jaundice present | | |
| **HEENT** | Anterior fontanelle open and flat, extraocular movements intact, no scleral icterus, no nasal discharge | | |
| **Neck** | Supple | | |
| **Lungs** | Clear to auscultation bilaterally | | |
| **Cardiovascular** | Regular rate and rhythm, normal S1/S2, no murmurs | | |
| **Abdomen** | Soft, nondistended, no organomegaly | | |
| **Neurological** | Normal tone, normal reflexes | | |
| **Skin** | Facial jaundice present, no rashes or lesions | | |
| **GU** | Normal male genitalia, testes descended bilaterally | | |
| **Psychiatric** | N/A | | |

| **Instructor Notes - Changes and CASE Branch Points** | | |
| --- | --- | --- |
| **Intervention / Time point** | **Change in Case** | **Additional Information** |
| If learner does not explain the reason for why an interpreter is needed… | Mom should insist on using friend or friend can insist on interpreting | This is because we want the learners to be aware of why an interpreter is needed (e.g., for legal purposes, for communication efficiency) |
| If learner clearly states why an interpreter is needed… | A certified interpreter may enter the encounter | We had the faculty observer cue the interpreter to enter the room at this step |
| If need for interpreter is not identified within 90 seconds… | Behavior of the mom and/or friend gets strange to help learner realize mistake | Examples: appearing more anxious or getting upset |
| If learner does not identify each person in each room… | Do not offer your name/role to learner | Some of our residents assumed the mom’s friend was the interpreter or was the parent. In this case, do not have the mom’s friend clarify that she is not the parent. |
| If learner does not maintain direct eye contact with the patient/caregiver… | Interpreter may redirect him/her to keep eye contact with patient/caregiver | The interpreter can simply state: “Please direct your eye contact with the parent or patient” |

**Ideal Scenario Flow**

The learner enters the room and sees mom holding baby instead of the baby being under the phototherapy lights. The resident should introduce him/herself and explain his/her role. The learner should identify the need for a certified interpreter and should clearly explain to the parent and friend in the room why it is important. The learner should perform the critical actions (e.g., maintain eye contact with parent, avoid medical jargon, etc) once the interpreter joins the encounter. After addressing mom’s questions and concerns, the learner should use teach back to ensure comprehension. The encounter should end afterward, with mom agreeing to phototherapy.

**Anticipated Management Mistakes**

- The learner relies on mom’s friend to interpret: We found that some of the learners assumed that the mom’s friend was an interpreter already waiting for them in the room. If the learner did not ask who each person was in the room, they often failed to recognize who was the parent in the encounter. This was avoided by having mom hold the baby from the beginning of the encounter.
- Uncertainty of explaining hyperbilirubinemia: We found that some learners were hesitant to explain hyperbilirubinemia if they were unsure of its pathophysiology. We also found that some learners thought that they were being graded on their medical knowledge, so we reminded them that this simulation focused more on communication skills.
