# Supplementary material for: Lost in Translation: An OSCE-Based Workshop for Helping Learners Navigate a Limited English Proficiency Patient Encounter
Source: MedEdPORTAL. 2021 Mar 17;17:11118. doi: 10.15766/mep_2374-8265.11118 (PMC7970641; doi:10.15766/mep_2374-8265.11118)
Supplement: Supplementary file 1 — Description of Workshop Components.docxChecklist.docxPreworkshop OSCE.docxPanel Discussion.docxWorking With Health Care Interpreters.pptxMap of Postworkshop OSCE.docxFacilitator Guide for Interactive Q&A.docxDebriefing.docxPostworkshop OSCE.docx [file mep_2374-8265.11118-s001.zip › D. Panel Discussion.docx]

**Appendix D: Panel Discussion**

We suggest allotting 30 minutes of time for this activity. Our panel comprised of 4 professionals from different backgrounds who work with the Limited English Proficiency population. We understand that your panel will be shaped by who is available at your institution. Potential panel members include a certified health interpreter, healthcare provider, social worker, a member of your diversity and inclusion office, or a patient or family representative. Panelists should have experience working with LEP patients and be passionate about providing care for this population.

A facilitator asked panelists the questions listed below, and audience members had the opportunity to ask additional questions. We also provide prompts to help with discussion (see bullets below).

**Guided questions:**

1. How did you choose your professional field?
   - *Please have panel members elaborate on motivation, experiences, or interesting anecdotes*
2. What barriers do you encounter?
   - *Please describe difficult situations and give examples (can be with either patients, colleagues, or medical professionals)*
   - *Are there specific cultural barriers you have witnessed (such as evil eye, or patriarchal vs matriarchal cultures?)*
3. Can you describe a time where a medical error occurred due to a language barrier?
   - *Please describe what type of medical error occurred and different causes (e.g., a cultural misunderstanding, not using an interpreter for the encounter, etc.)*
4. Can you describe a personal “win” where you felt like you made a big difference for a LEP patient?
   - *Please describe the situation and why you felt like you made a difference*
5. What is one piece of advice you would like to impart on our learners today?
   - *Examples include tips and tricks, give a lesson you learned from a challenge you encountered, reminder to “do no harm”*
