# Supplementary material for: Lost in Translation: An OSCE-Based Workshop for Helping Learners Navigate a Limited English Proficiency Patient Encounter
Source: MedEdPORTAL. 2021 Mar 17;17:11118. doi: 10.15766/mep_2374-8265.11118 (PMC7970641; doi:10.15766/mep_2374-8265.11118)
Supplement: Supplementary file 1 — Description of Workshop Components.docxChecklist.docxPreworkshop OSCE.docxPanel Discussion.docxWorking With Health Care Interpreters.pptxMap of Postworkshop OSCE.docxFacilitator Guide for Interactive Q&A.docxDebriefing.docxPostworkshop OSCE.docx [file mep_2374-8265.11118-s001.zip › E. Working With Health Care Interpreters.pptx]

## Slide 1
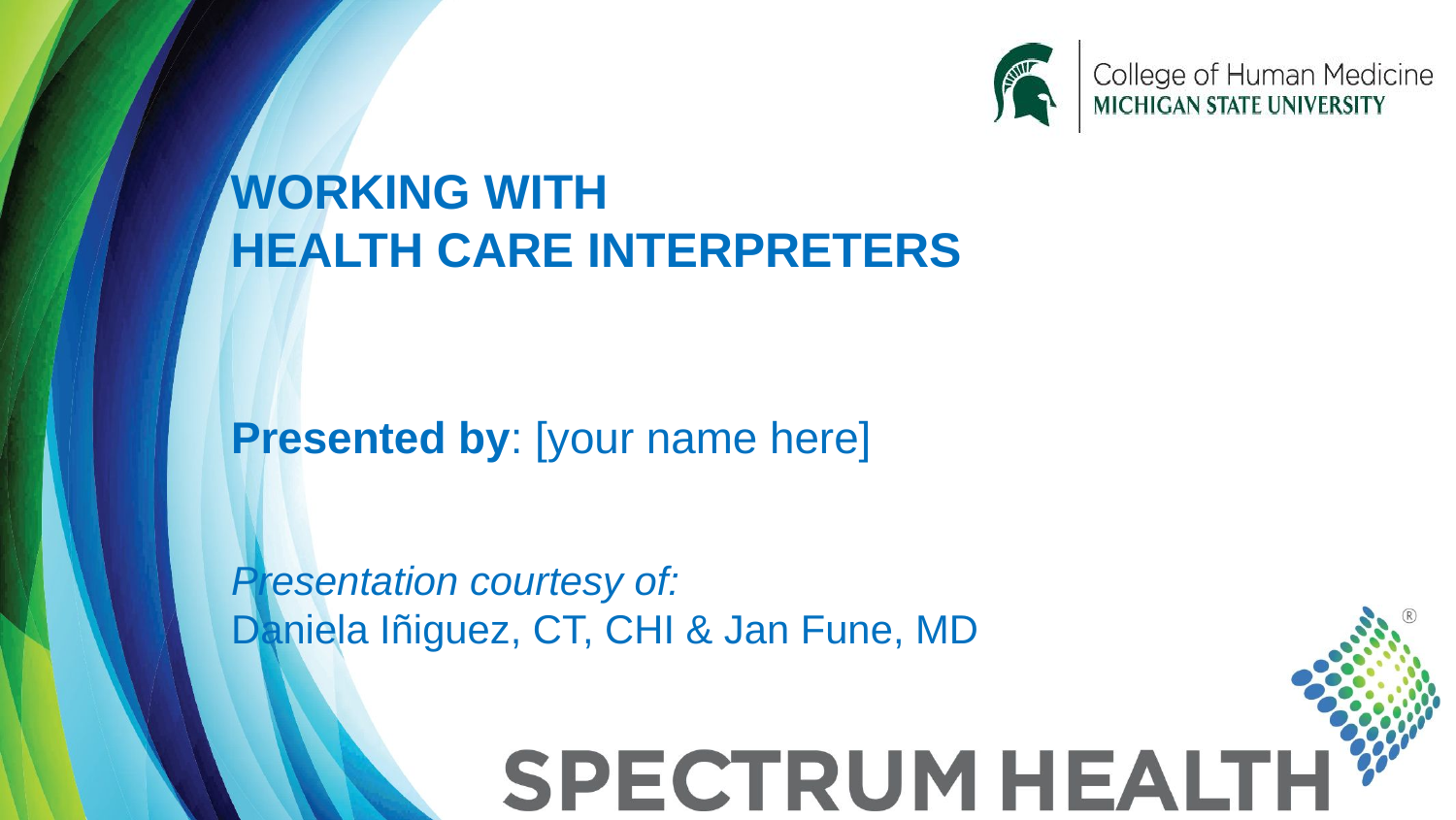

# WORKING WITH HEALTH CARE INTERPRETERS
Presented by: [your name here]
Presentation courtesy of:
Daniela Iñiguez, CT, CHI & Jan Fune, MD

## Slide 2
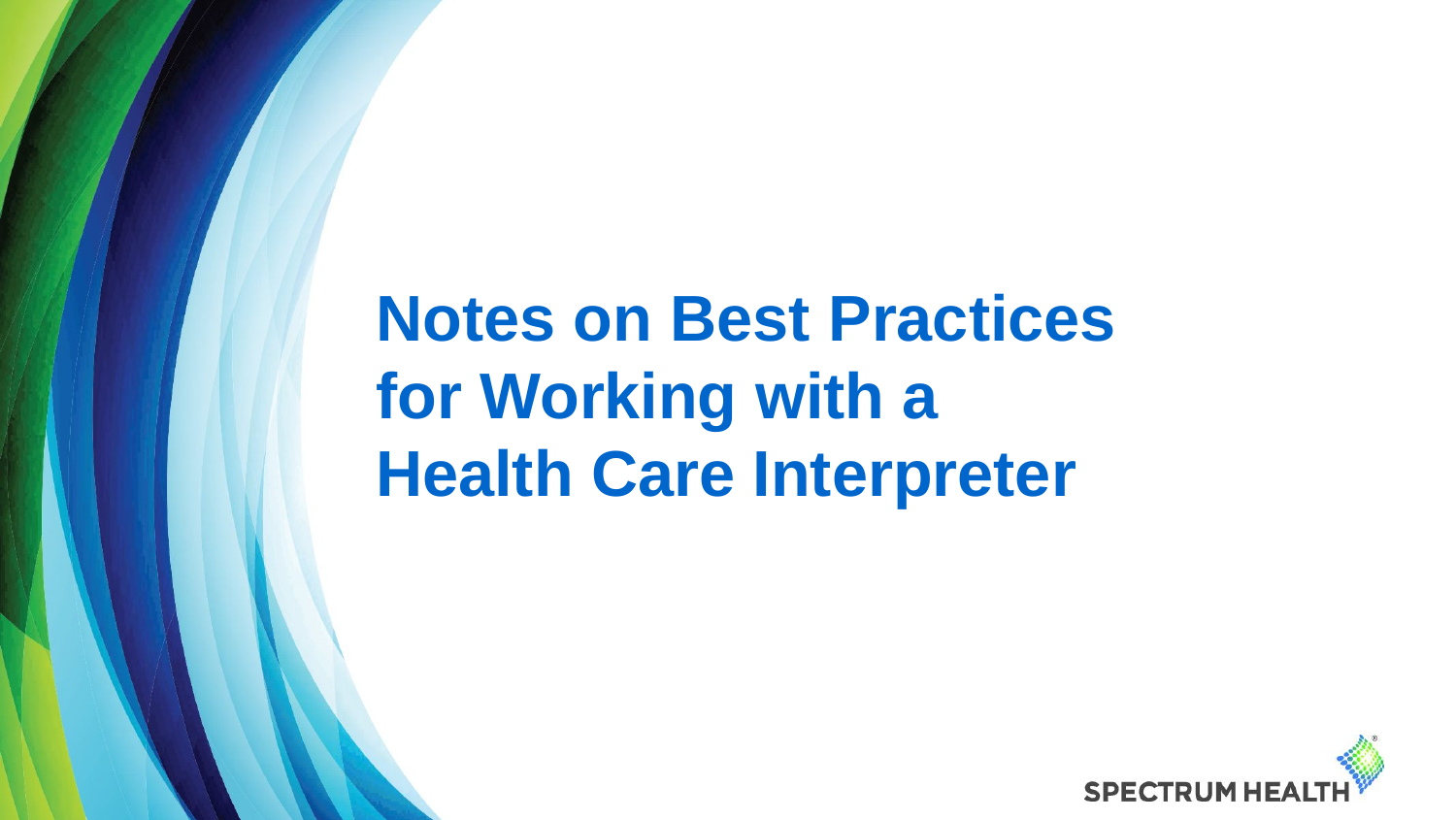

# Notes on Best Practices for Working with a Health Care Interpreter

## Slide 3
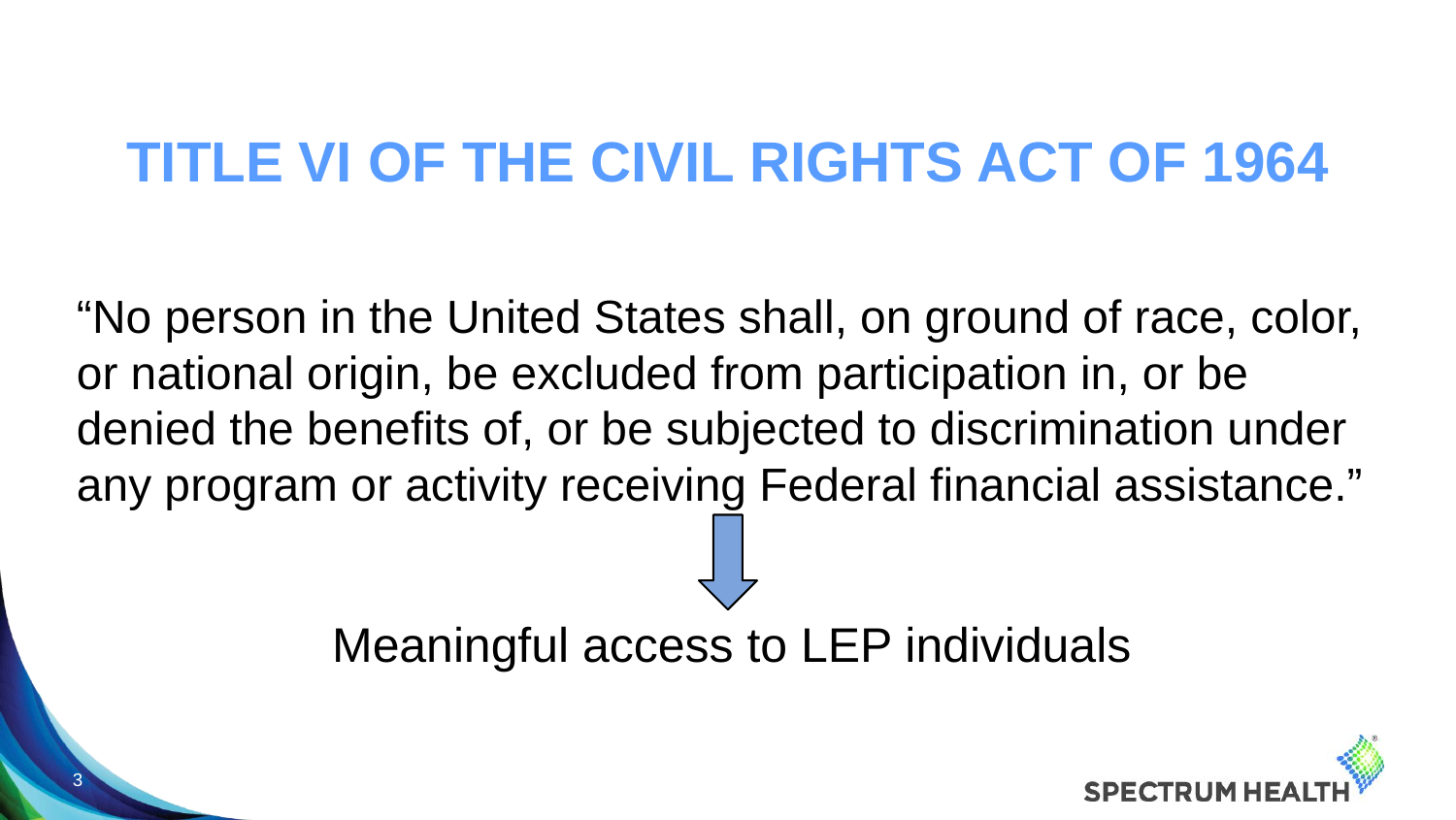

# Title VI of the Civil Rights Act OF 1964
“No person in the United States shall, on ground of race, color, or national origin, be excluded from participation in, or be denied the benefits of, or be subjected to discrimination under any program or activity receiving Federal financial assistance.”
Meaningful access to LEP individuals
3

## Slide 4
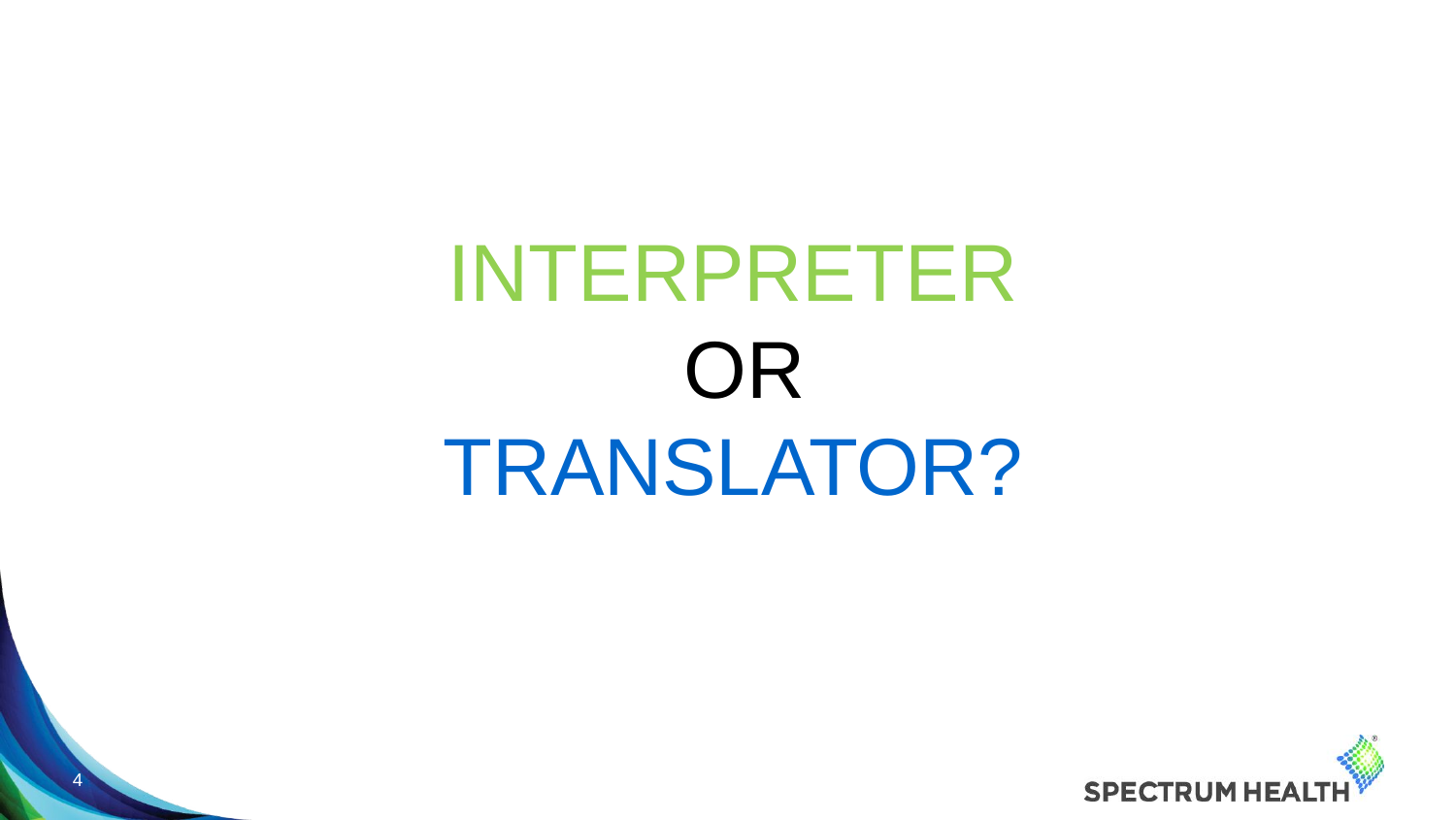

INTERPRETER
 OR
TRANSLATOR?
4

## Slide 5
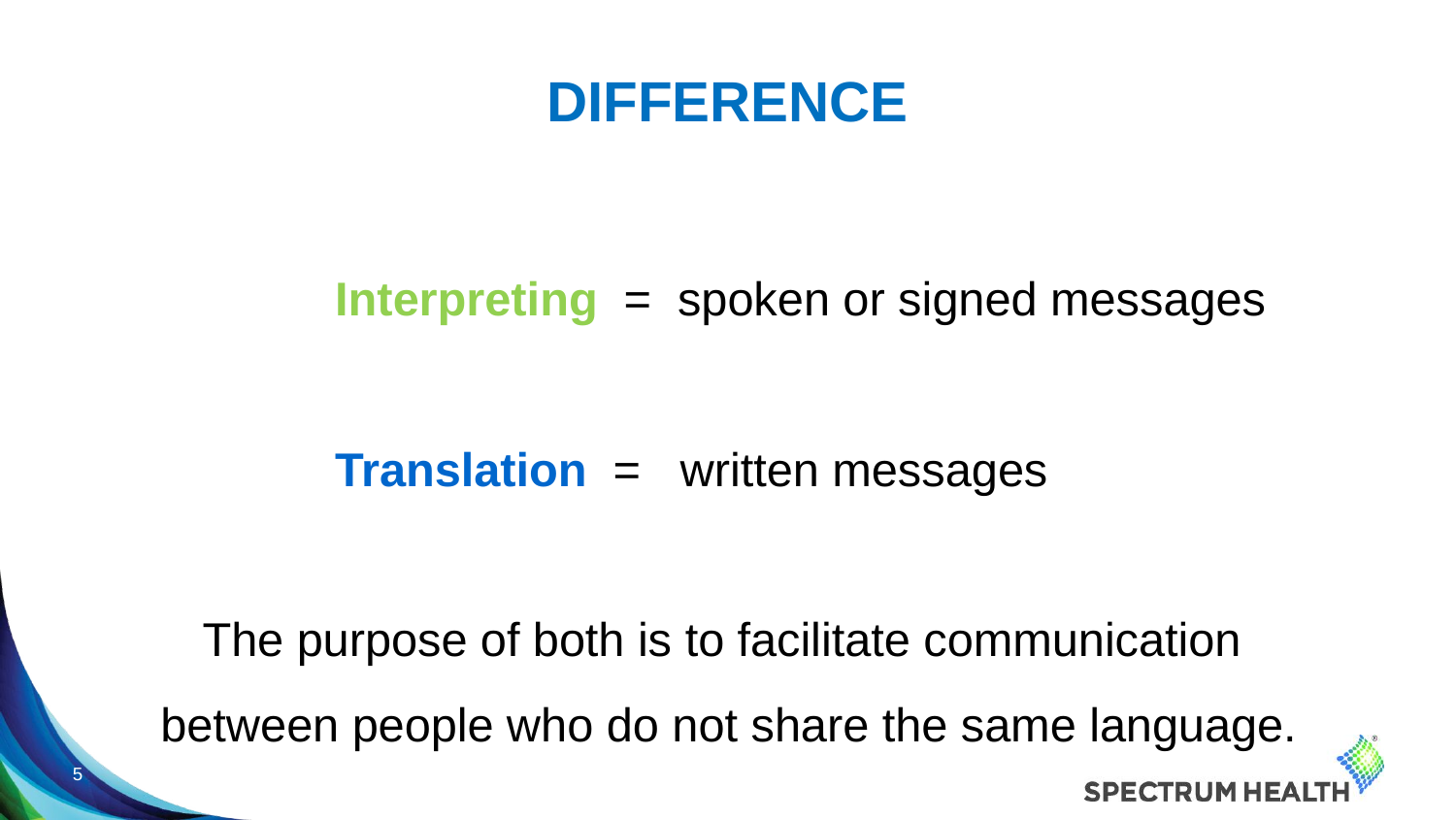

# DIFFERENCE
		Interpreting = spoken or signed messages
		Translation = written messages
The purpose of both is to facilitate communication
between people who do not share the same language.
5

## Slide 6
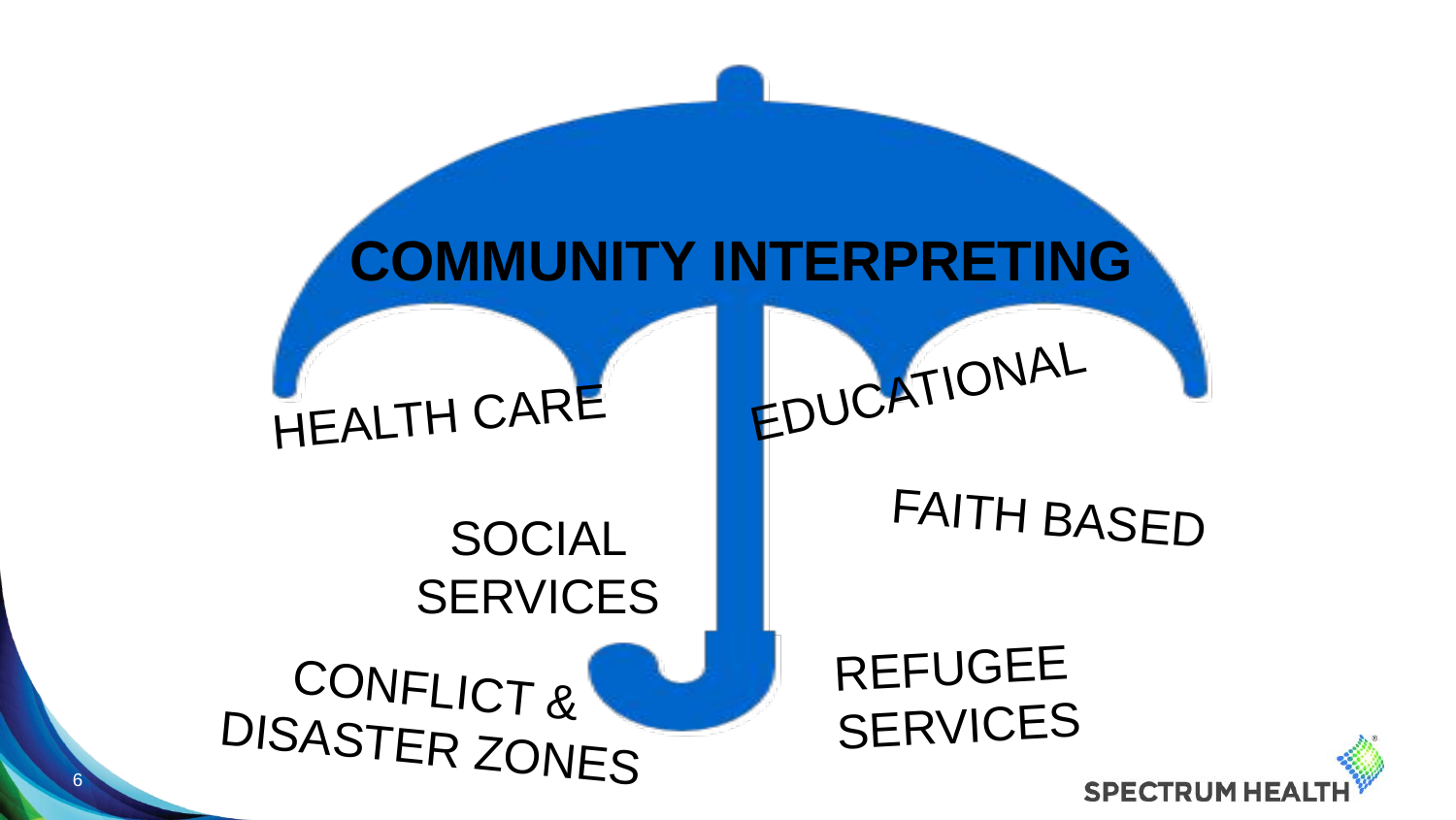

COMMUNITY INTERPRETING
EDUCATIONAL
HEALTH CARE
FAITH BASED
SOCIAL SERVICES
REFUGEE SERVICES
CONFLICT & DISASTER ZONES
6

## Slide 7
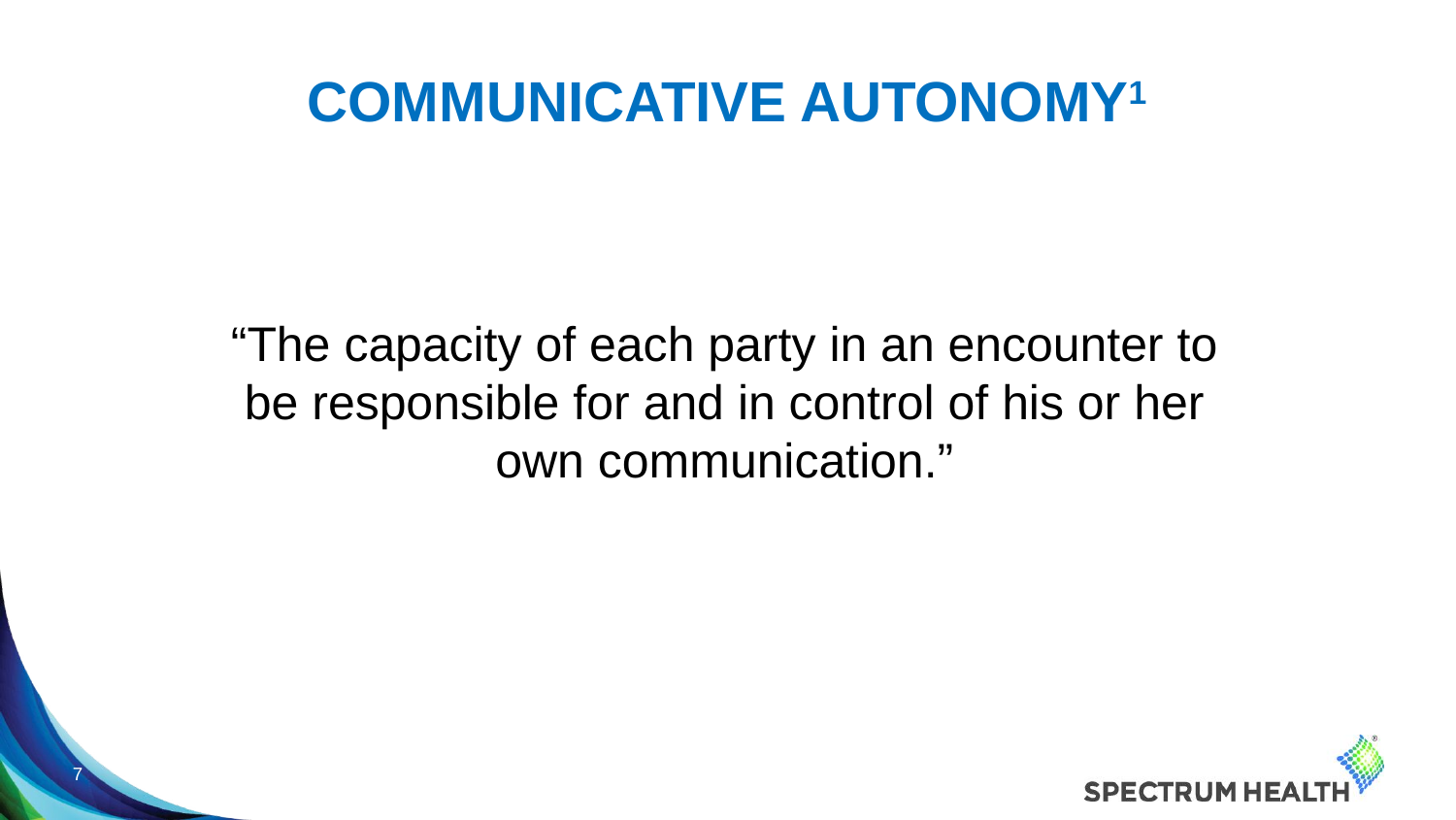

# COMMUNICATIVE AUTONOMY1
“The capacity of each party in an encounter to be responsible for and in control of his or her own communication.”
7

## Slide 8
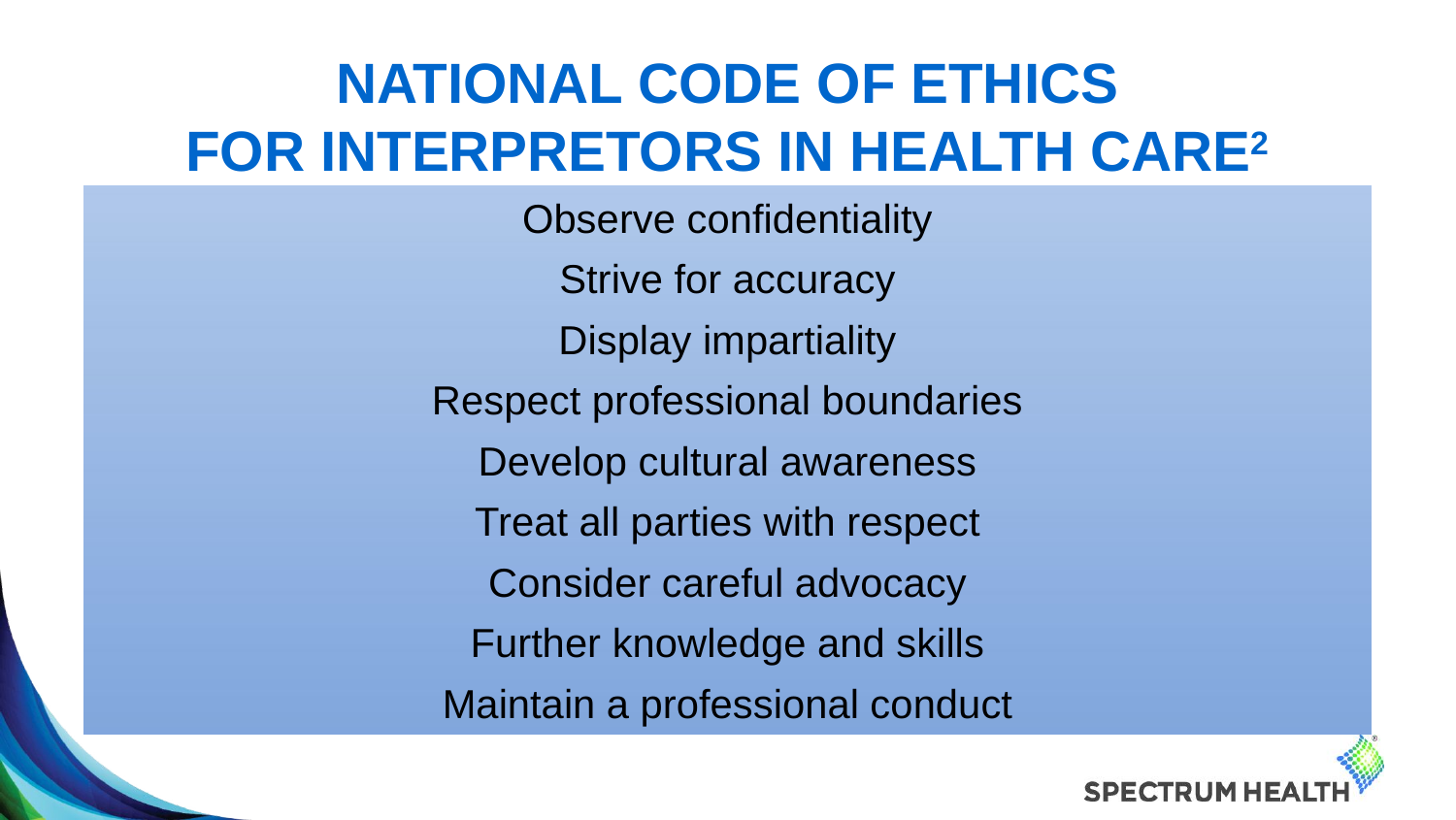

# NATIONAL CODE OF ETHICS FOR INTERPRETORS IN HEALTH CARE2
Observe confidentiality
Strive for accuracy
Display impartiality
Respect professional boundaries
Develop cultural awareness
Treat all parties with respect
Consider careful advocacy
Further knowledge and skills
Maintain a professional conduct

## Slide 9
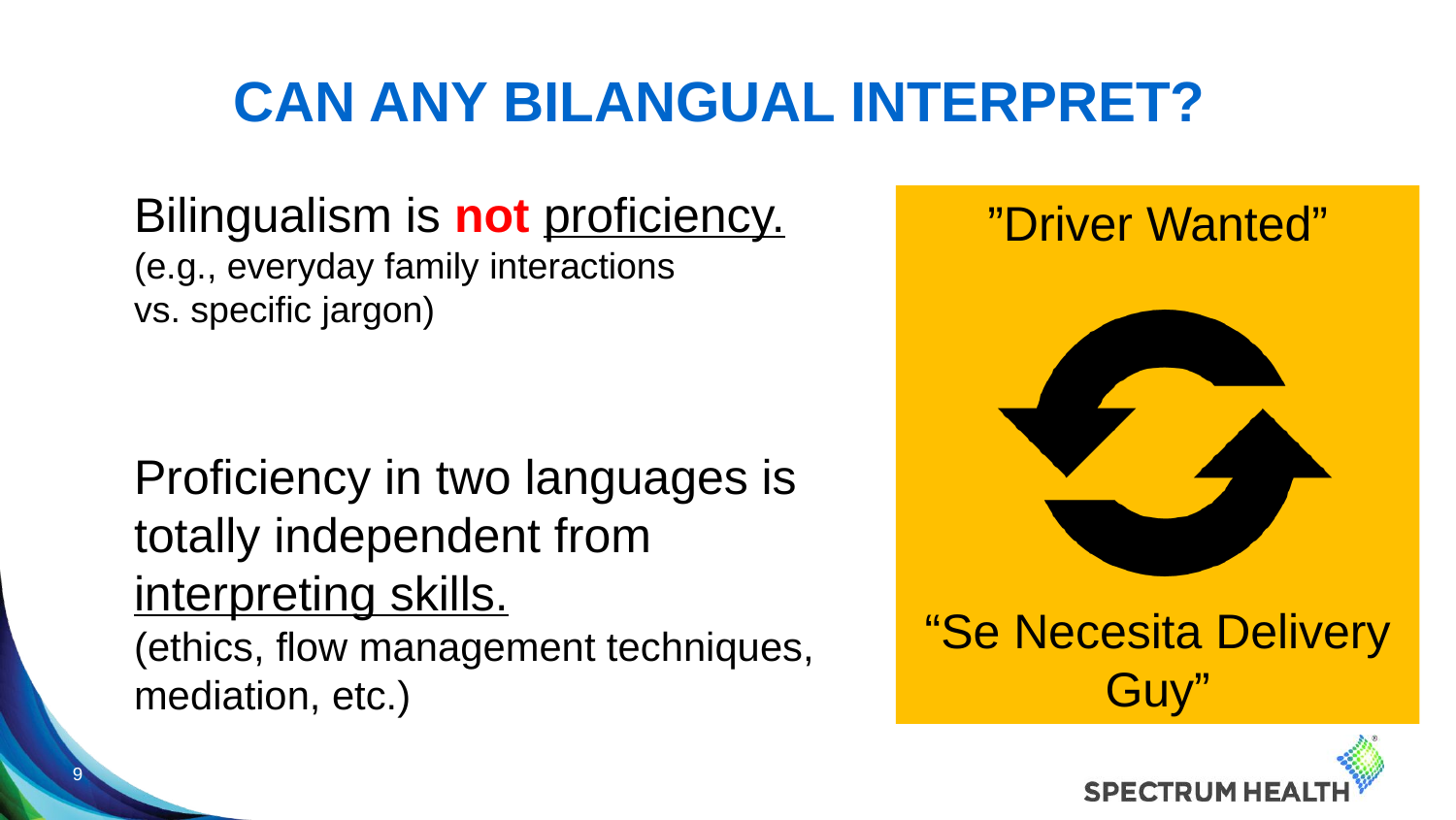

# CAN ANY BILANGUAL INTERPRET?
Bilingualism is not proficiency.
(e.g., everyday family interactions
vs. specific jargon)
Proficiency in two languages is totally independent from interpreting skills.
(ethics, flow management techniques, mediation, etc.)
”Driver Wanted”
“Se Necesita Delivery Guy”
9

## Slide 10
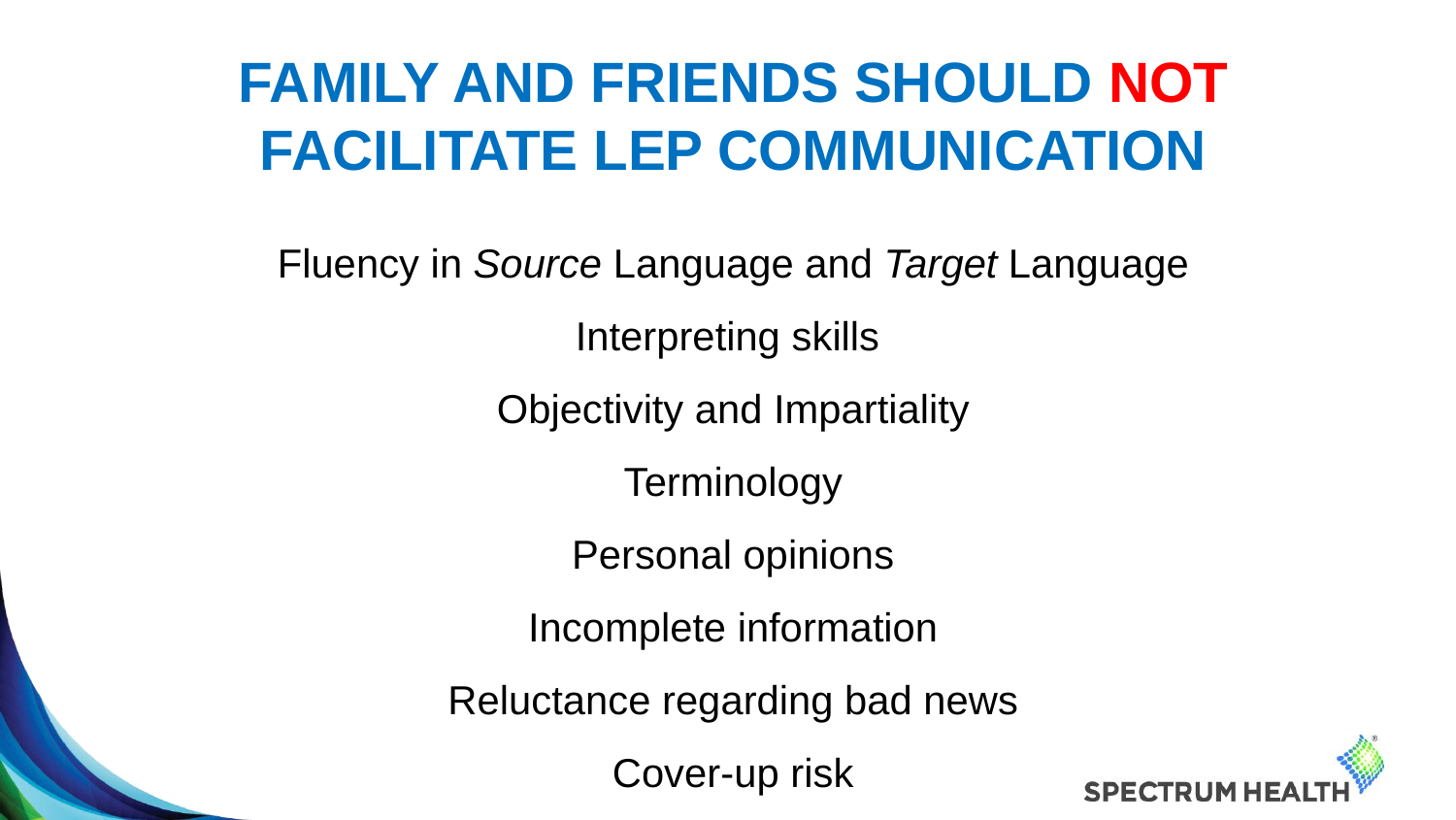

# FAMILY AND FRIENDS SHOULD NOT FACILITATE LEP COMMUNICATION
Fluency in Source Language and Target Language
Interpreting skills
Objectivity and Impartiality
Terminology
Personal opinions
Incomplete information
Reluctance regarding bad news
Cover-up risk

## Slide 11
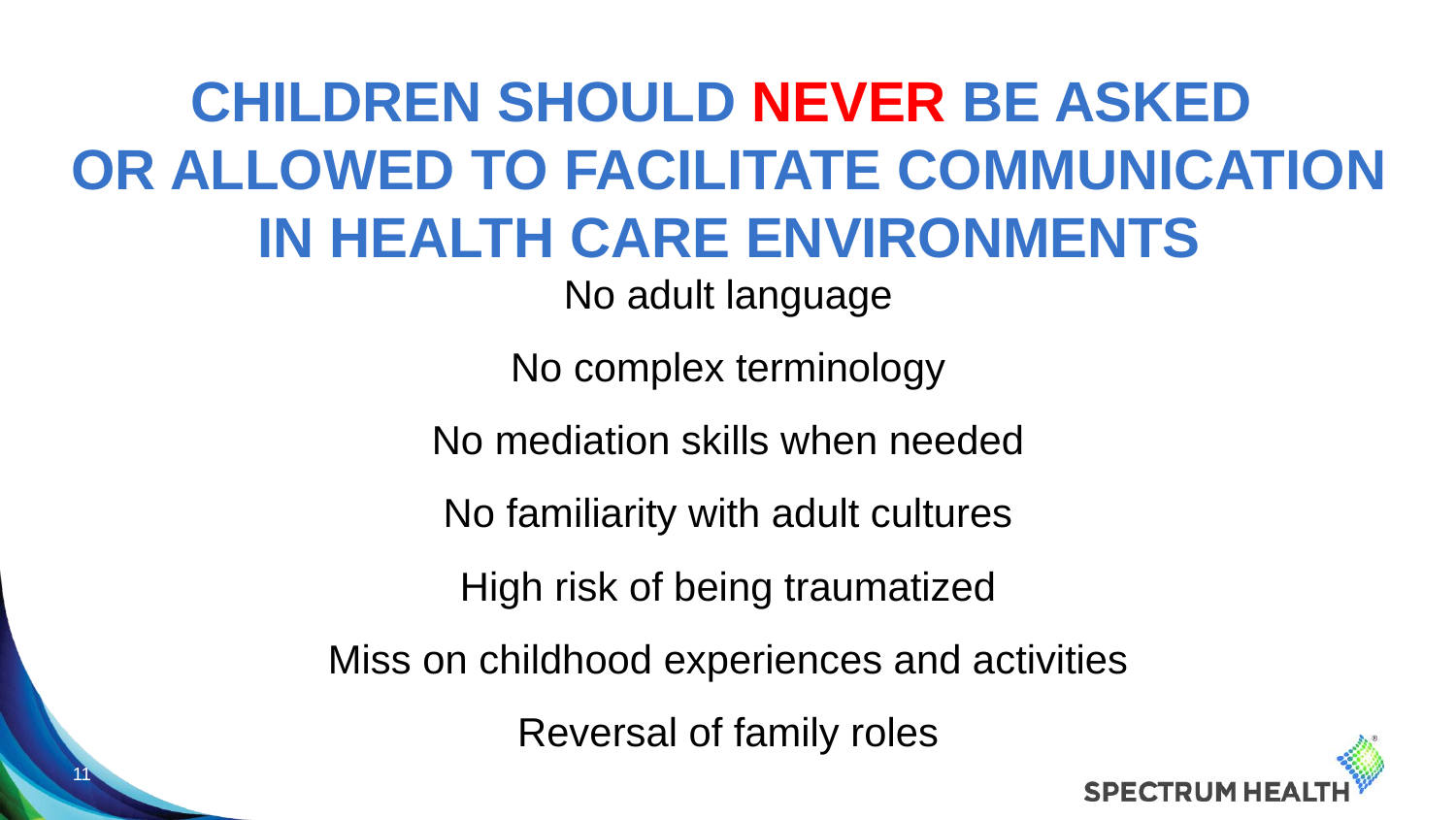

# CHILDREN SHOULD NEVER BE ASKED OR ALLOWED TO FACILITATE COMMUNICATION IN HEALTH CARE ENVIRONMENTS
No adult language
No complex terminology
No mediation skills when needed
No familiarity with adult cultures
High risk of being traumatized
Miss on childhood experiences and activities
Reversal of family roles
11

## Slide 12
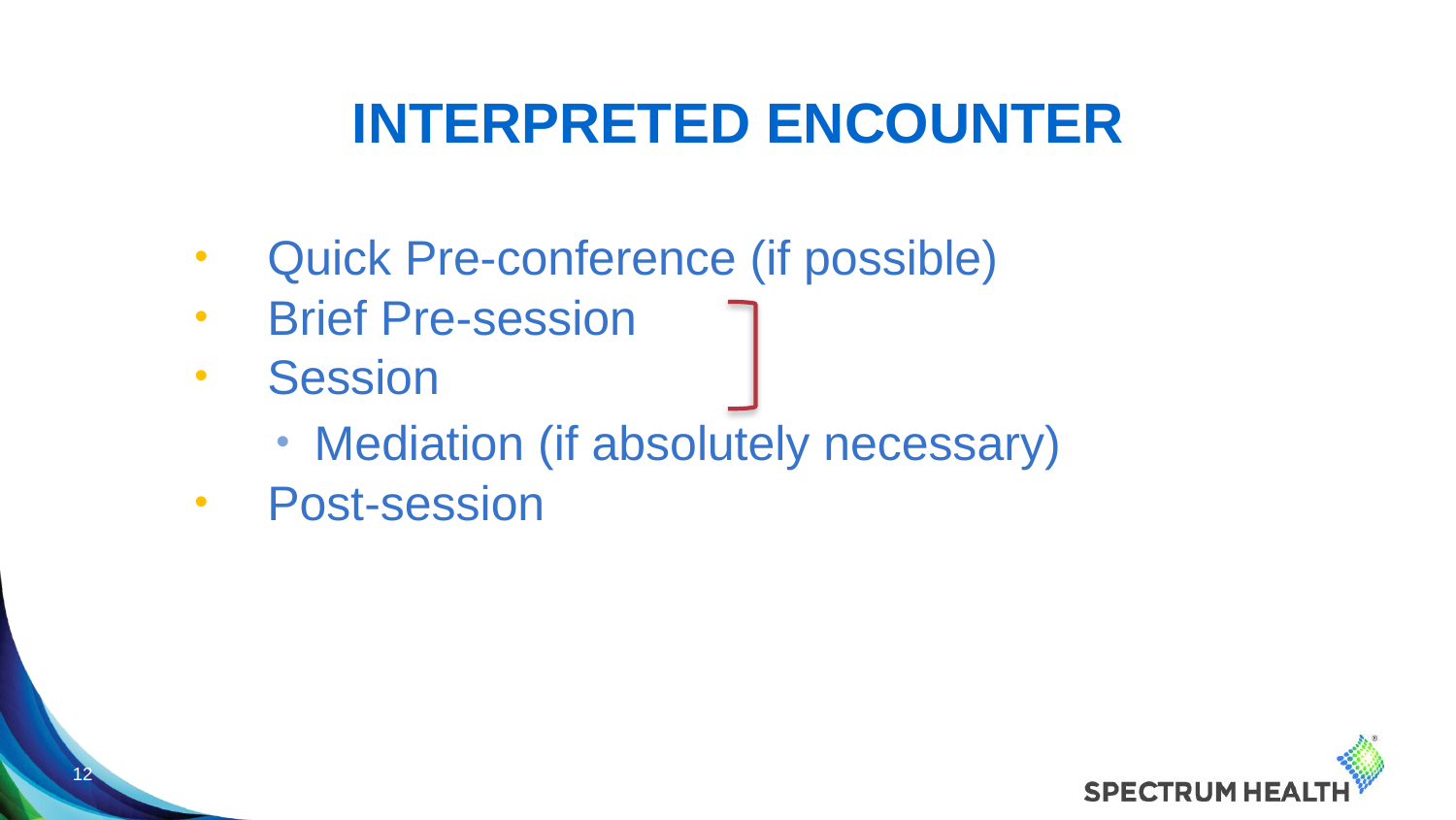

# INTERPRETED ENCOUNTER
Quick Pre-conference (if possible)
Brief Pre-session
Session
 Mediation (if absolutely necessary)
Post-session
12

## Slide 13
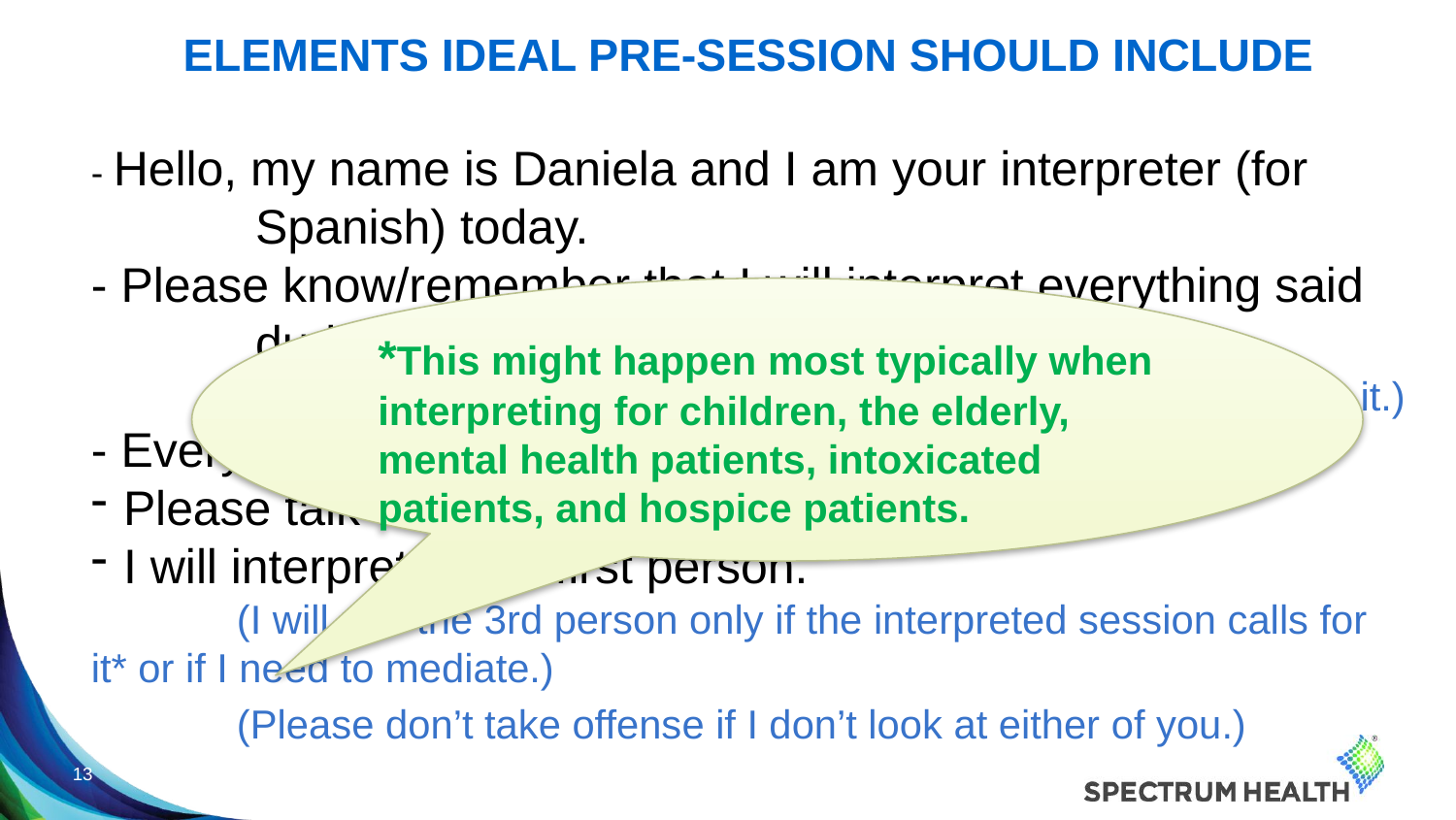

# ELEMENTS IDEAL PRE-SESSION SHOULD INCLUDE
- Hello, my name is Daniela and I am your interpreter (for 	Spanish) today.
- Please know/remember that I will interpret everything said 	during this encounter.
 	(If you don’t want me to interpret something, please don’t say it.)
- Everything said will remain confidential.
 Please talk directly to each other and not to me.
 I will interpret in the first person.
	(I will use the 3rd person only if the interpreted session calls for 	it* or if I need to mediate.)
	(Please don’t take offense if I don’t look at either of you.)
*This might happen most typically when interpreting for children, the elderly, mental health patients, intoxicated patients, and hospice patients.
13

## Slide 14
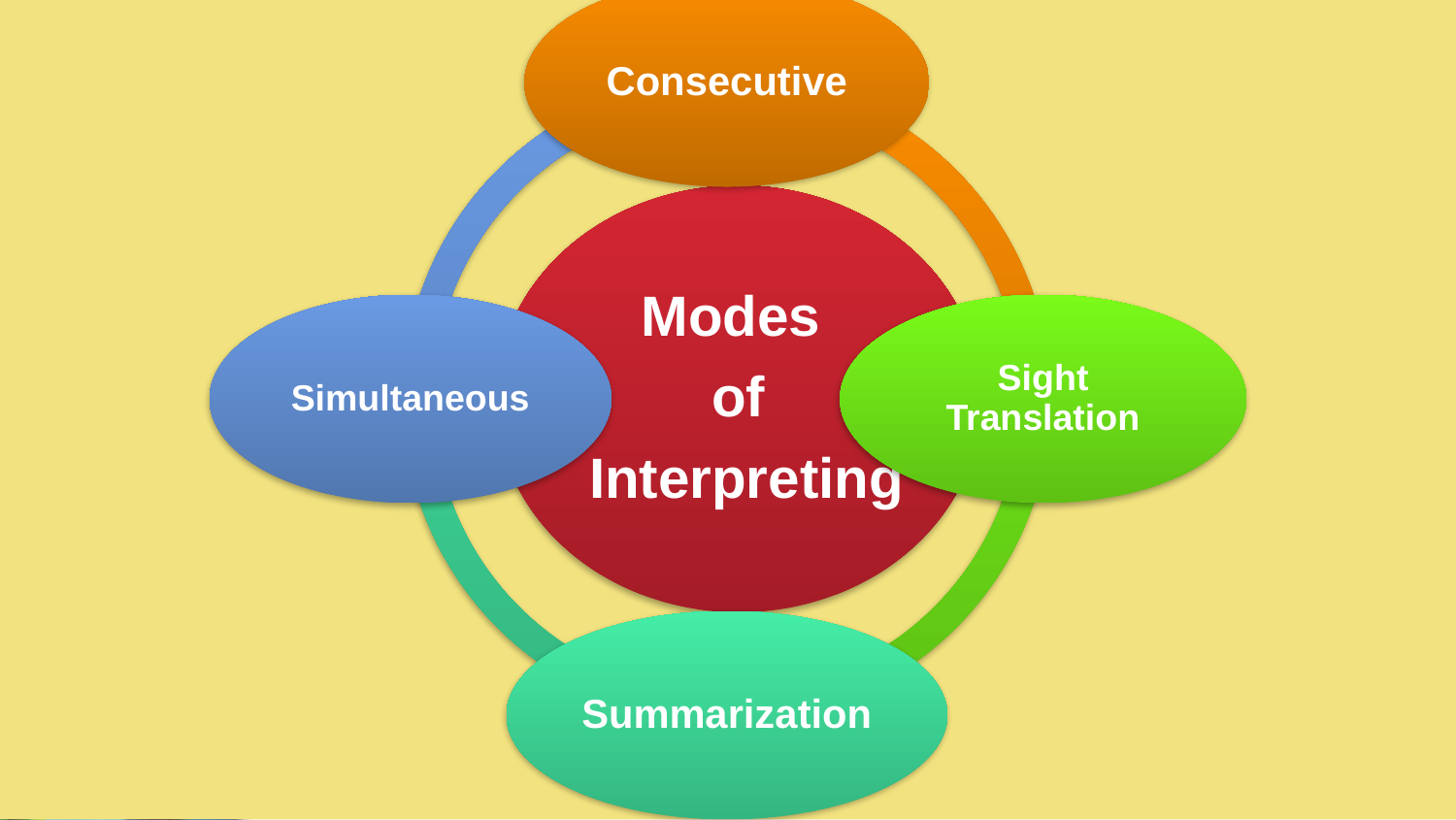

14

## Slide 15
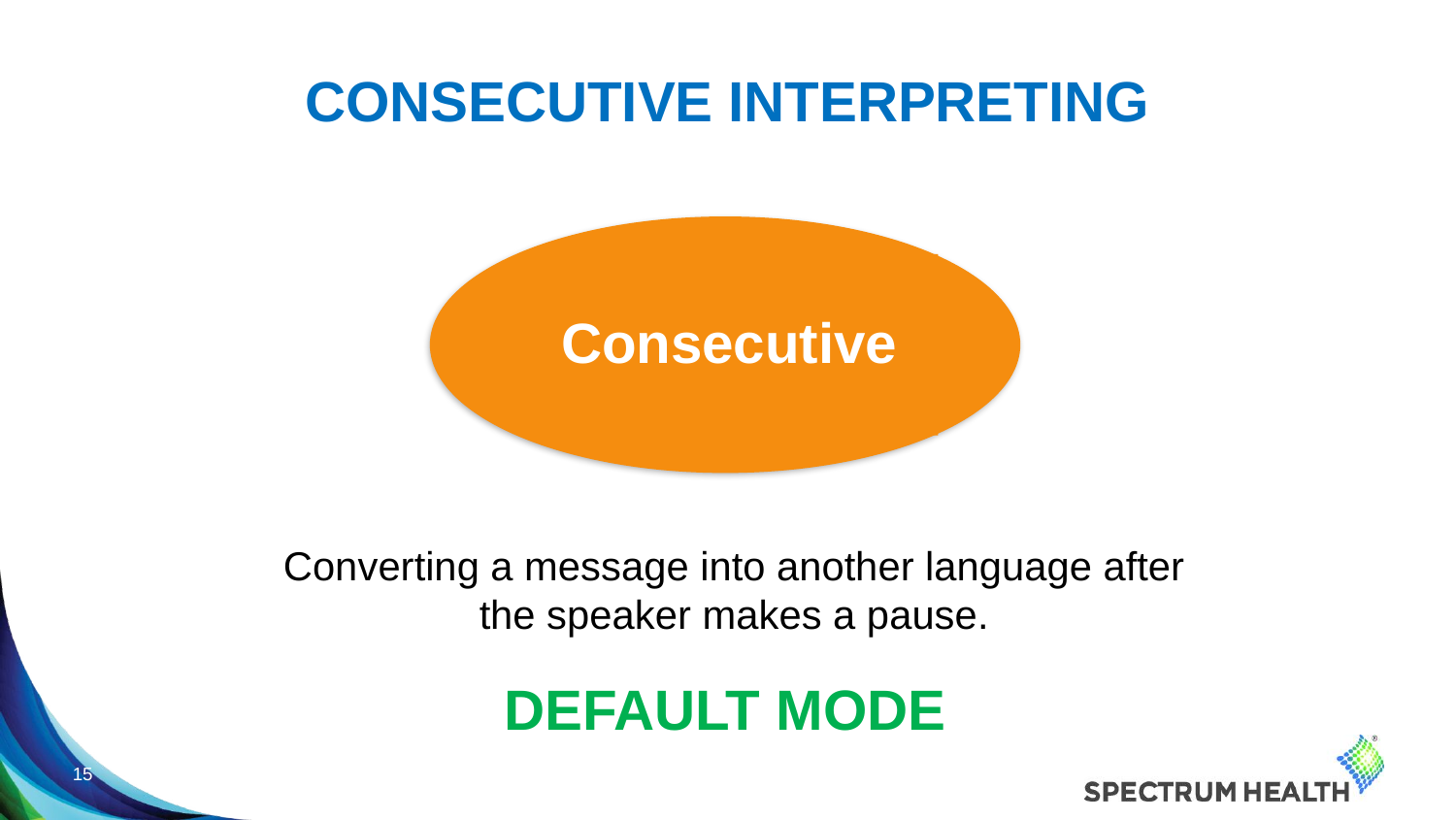

# CONSECUTIVE INTERPRETING
Consecutive
Converting a message into another language after the speaker makes a pause.
Default Mode
15

## Slide 16
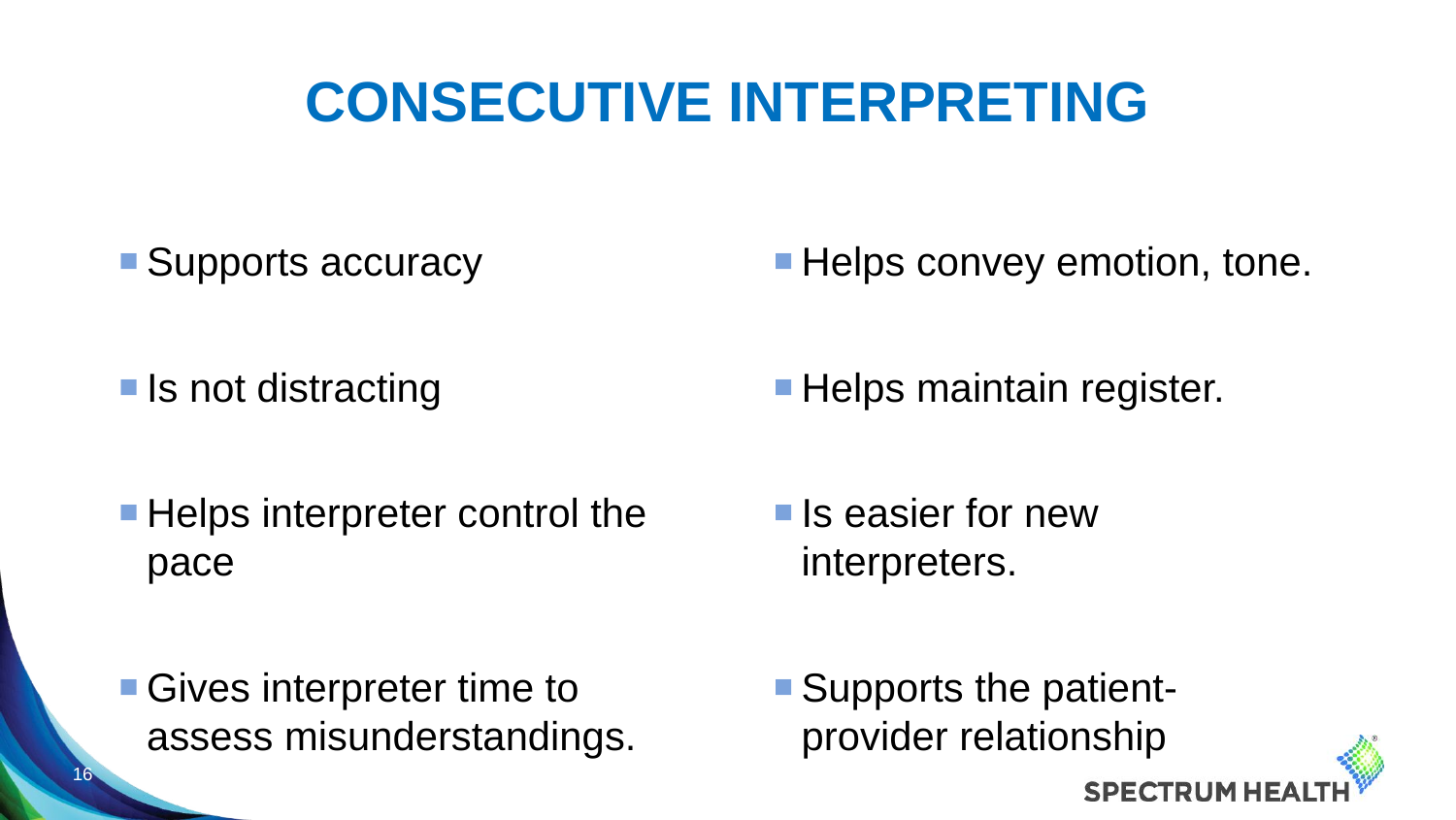

# CONSECUTIVE INTERPRETING
Supports accuracy
Is not distracting
Helps interpreter control the pace
Gives interpreter time to assess misunderstandings.
Helps convey emotion, tone.
Helps maintain register.
Is easier for new interpreters.
Supports the patient-provider relationship
16

## Slide 17
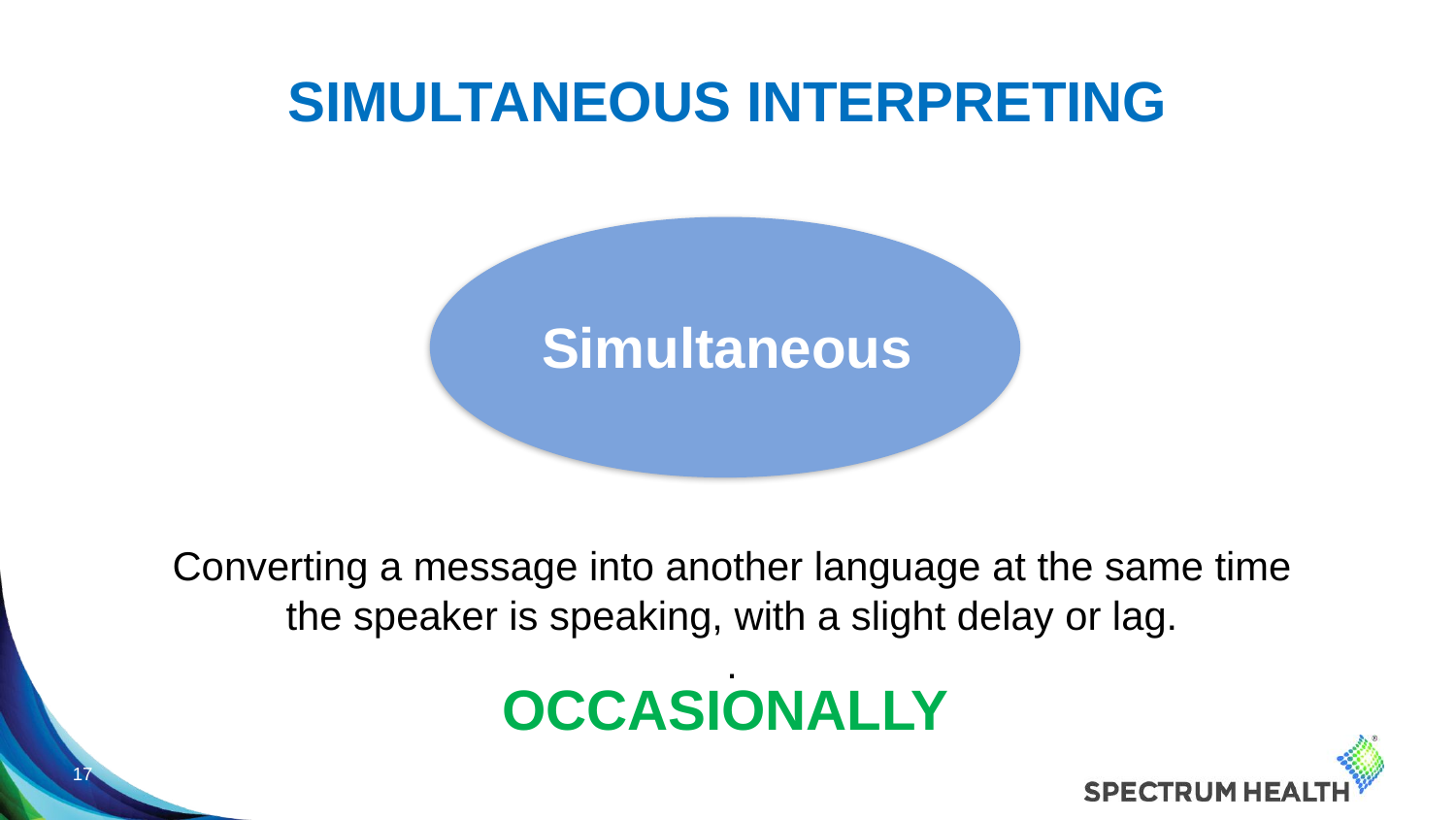

# SIMULTANEOUS INTERPRETING
Simultaneous
Converting a message into another language at the same time the speaker is speaking, with a slight delay or lag.
.
OCCASIONALLY
17

## Slide 18
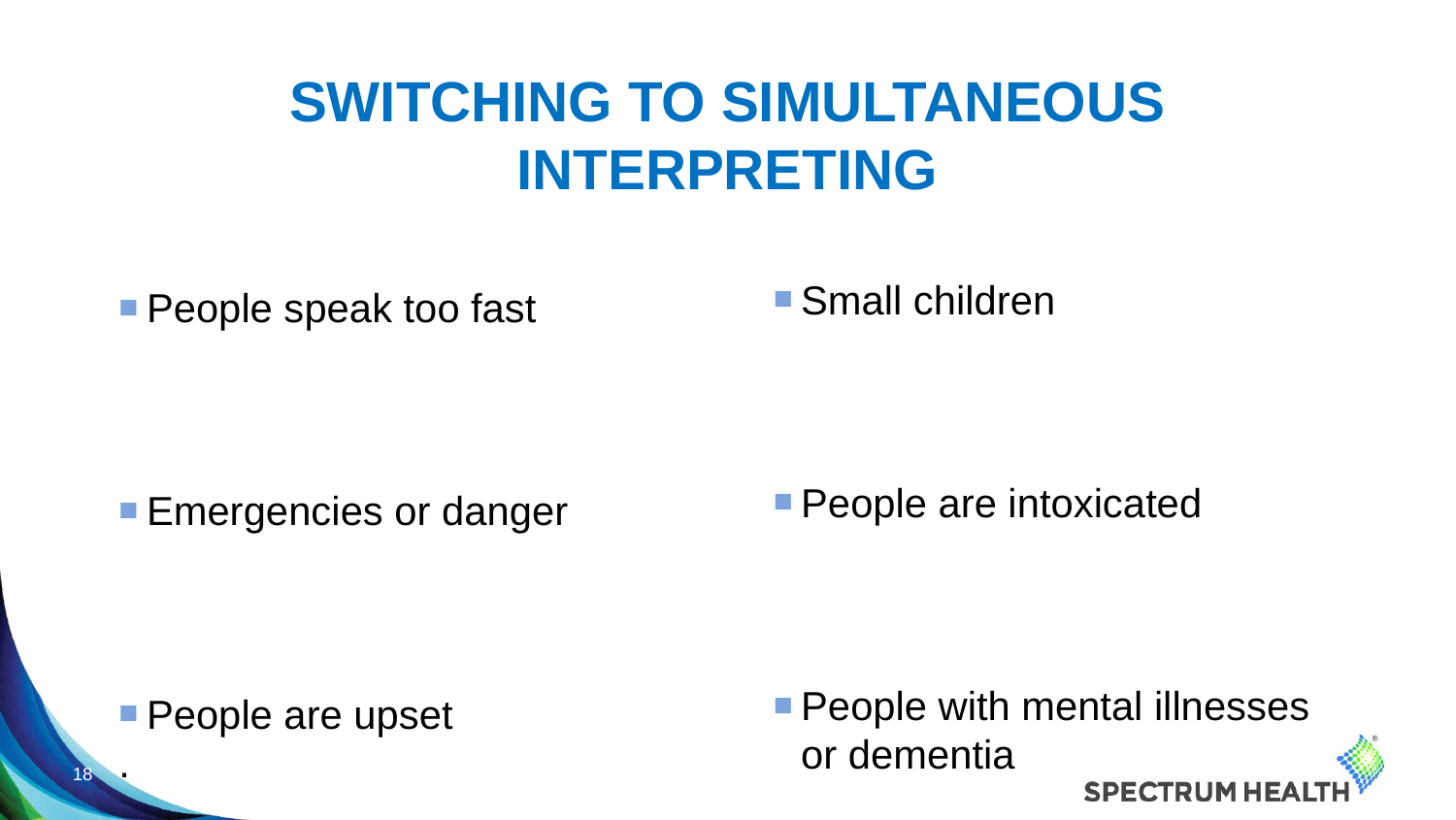

# SWITCHING TO SIMULTANEOUS INTERPRETING
Small children
People are intoxicated
People with mental illnesses or dementia
People speak too fast
Emergencies or danger
People are upset
.
18

## Slide 19
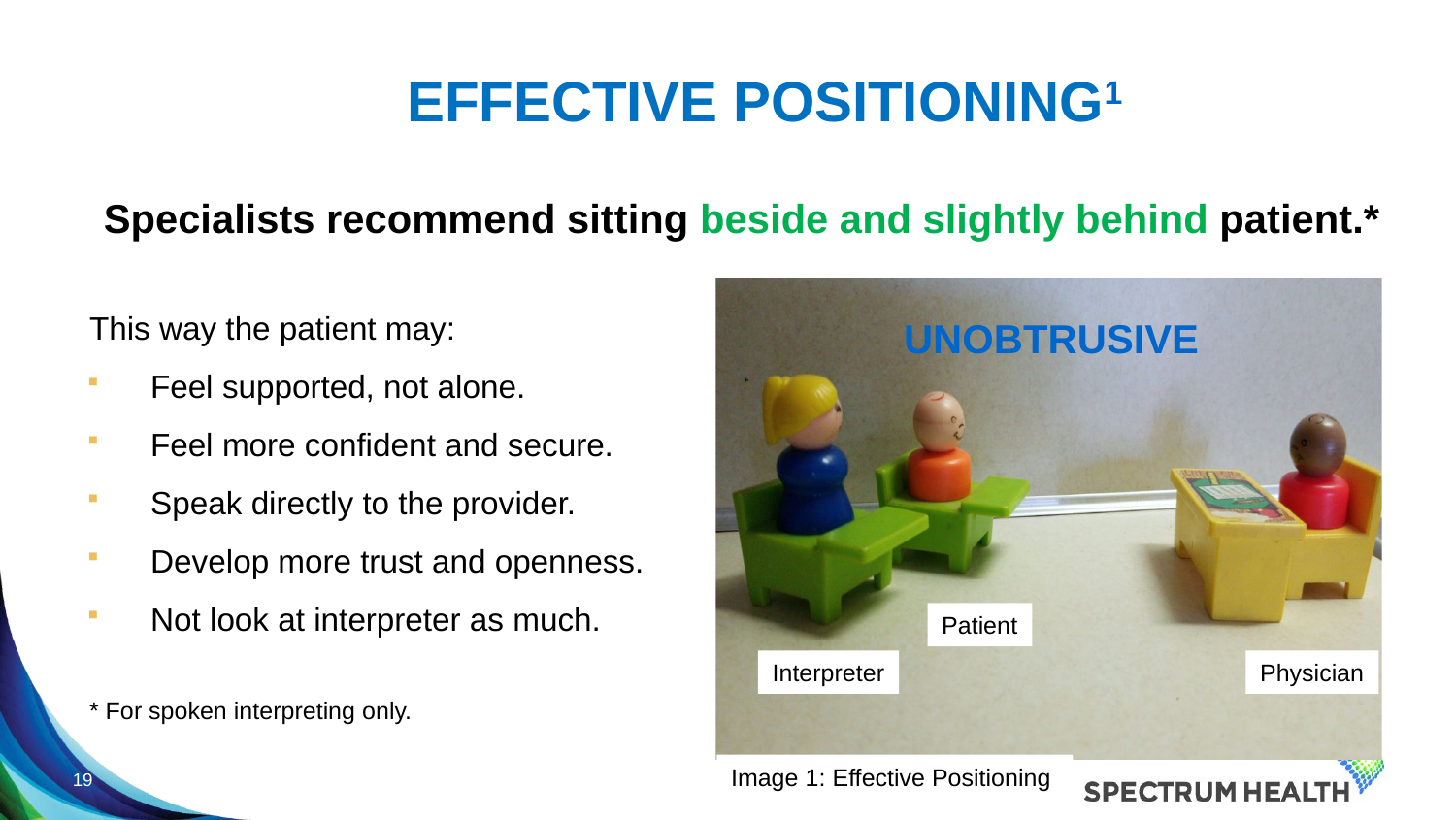

# EFFECTIVE POSITIONING1
Specialists recommend sitting beside and slightly behind patient.*
UNOBTRUSIVE
This way the patient may:
 Feel supported, not alone.
 Feel more confident and secure.
 Speak directly to the provider.
 Develop more trust and openness.
 Not look at interpreter as much.
* For spoken interpreting only.
Patient
Physician
Interpreter
Image 1: Effective Positioning
19

## Slide 20
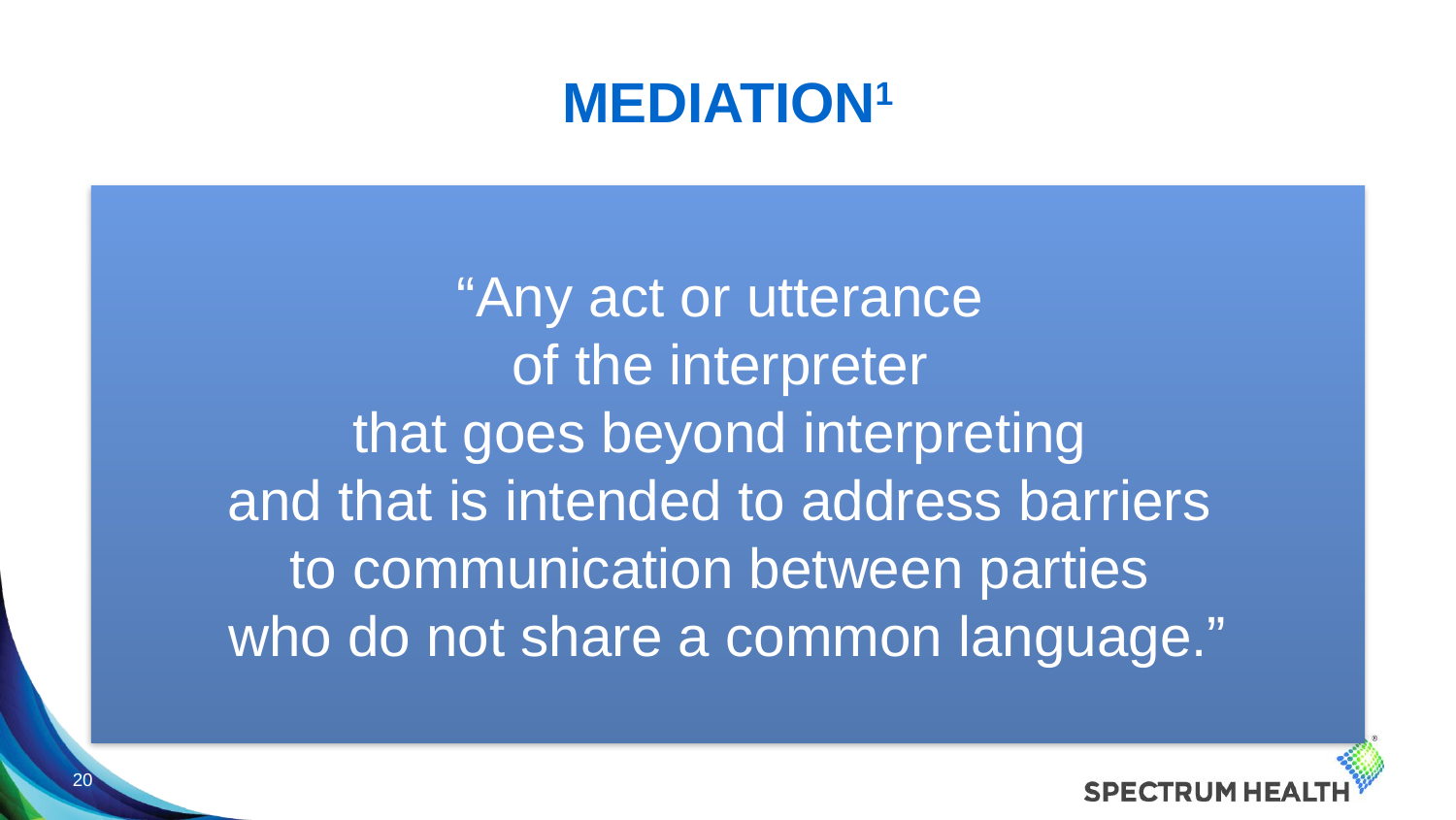

MEDIATION1
“Any act or utterance
of the interpreter
that goes beyond interpreting
and that is intended to address barriers
to communication between parties
who do not share a common language.”
20

## Slide 21
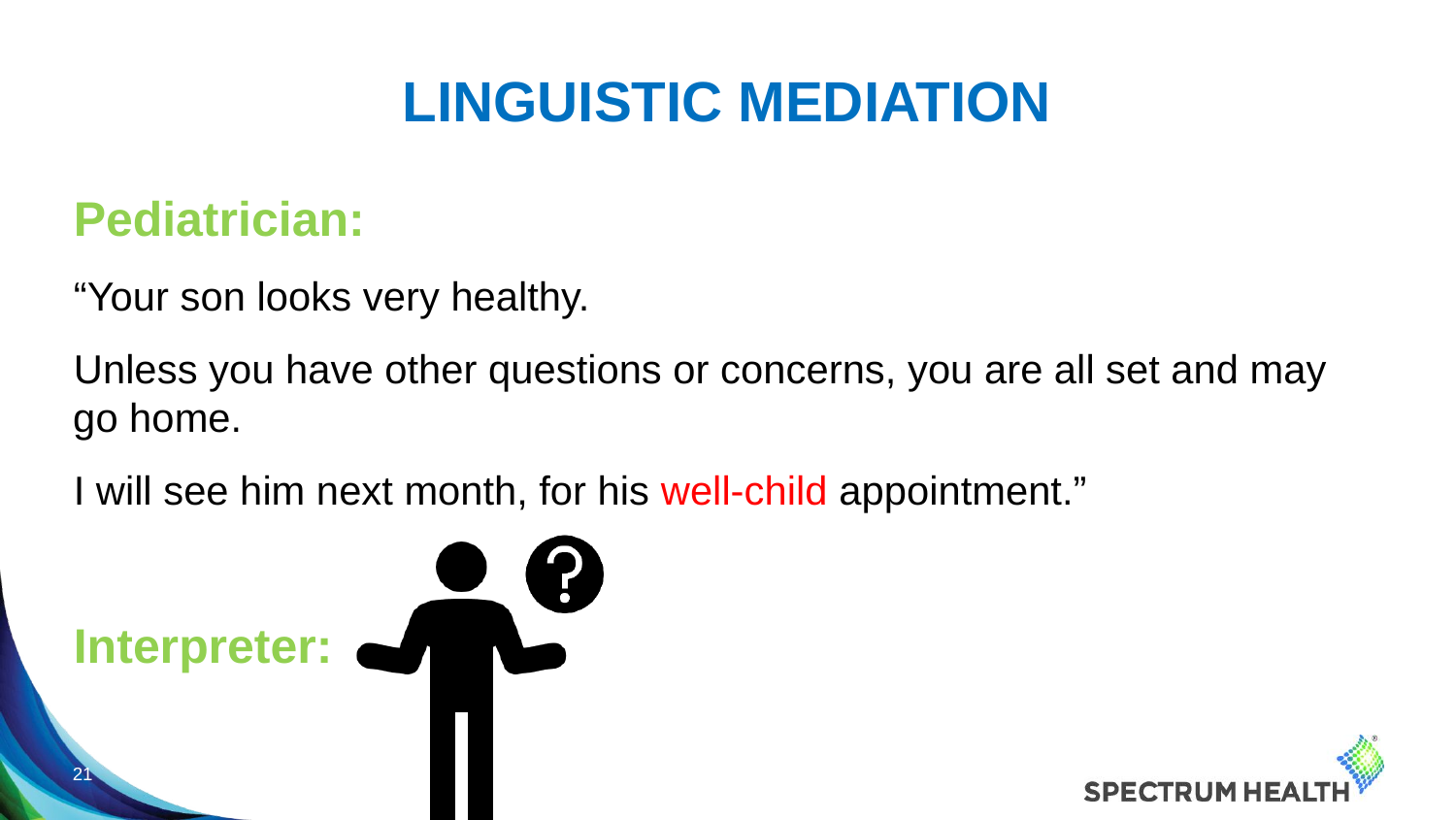

# LINGUISTIC MEDIATION
Pediatrician:
“Your son looks very healthy.
Unless you have other questions or concerns, you are all set and may go home.
I will see him next month, for his well-child appointment.”
Interpreter:
21

## Slide 22
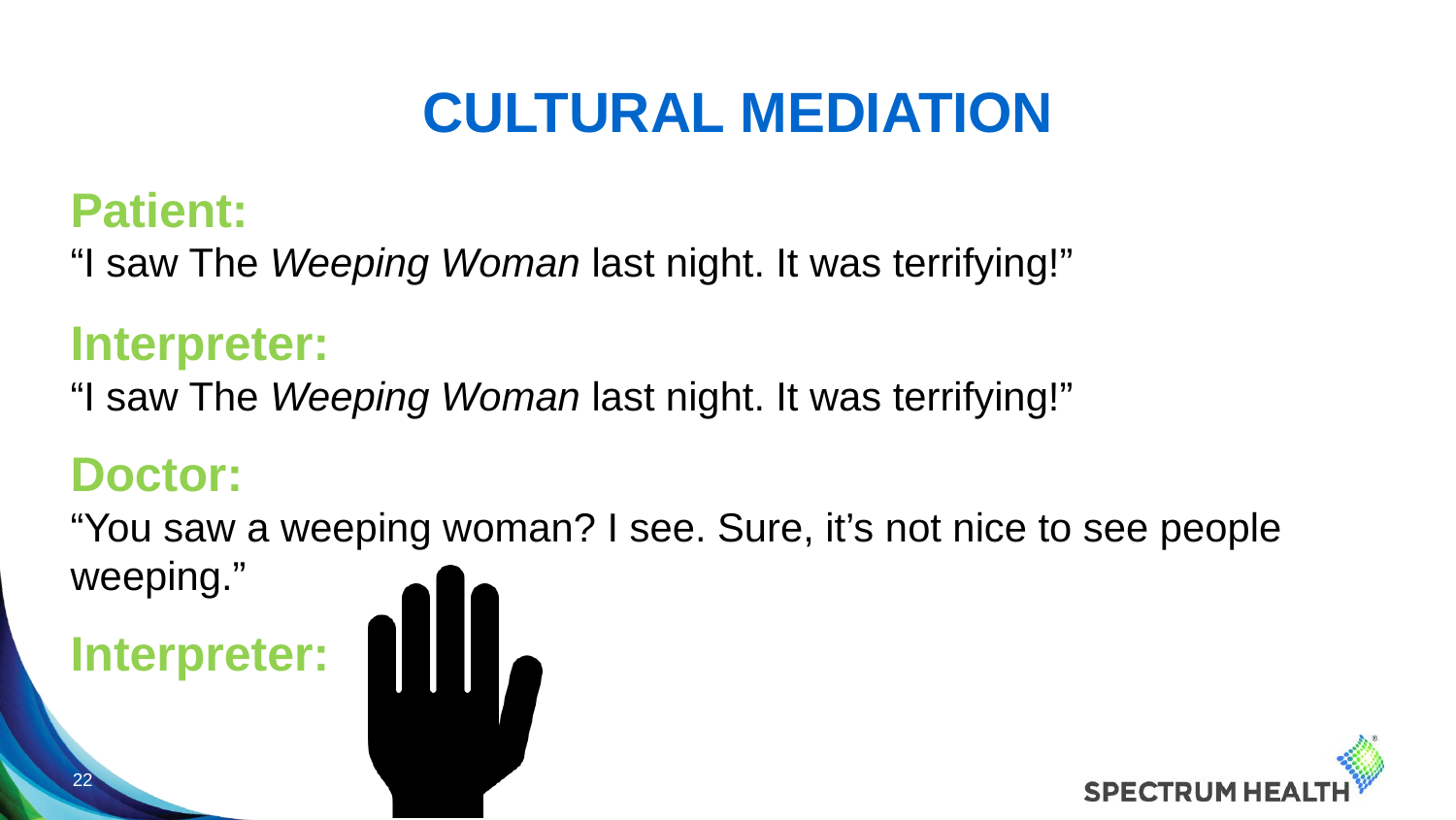

CULTURAL MEDIATION
Patient:
“I saw The Weeping Woman last night. It was terrifying!”
Interpreter:
“I saw The Weeping Woman last night. It was terrifying!”
Doctor:
“You saw a weeping woman? I see. Sure, it’s not nice to see people weeping.”
Interpreter:
22

## Slide 23
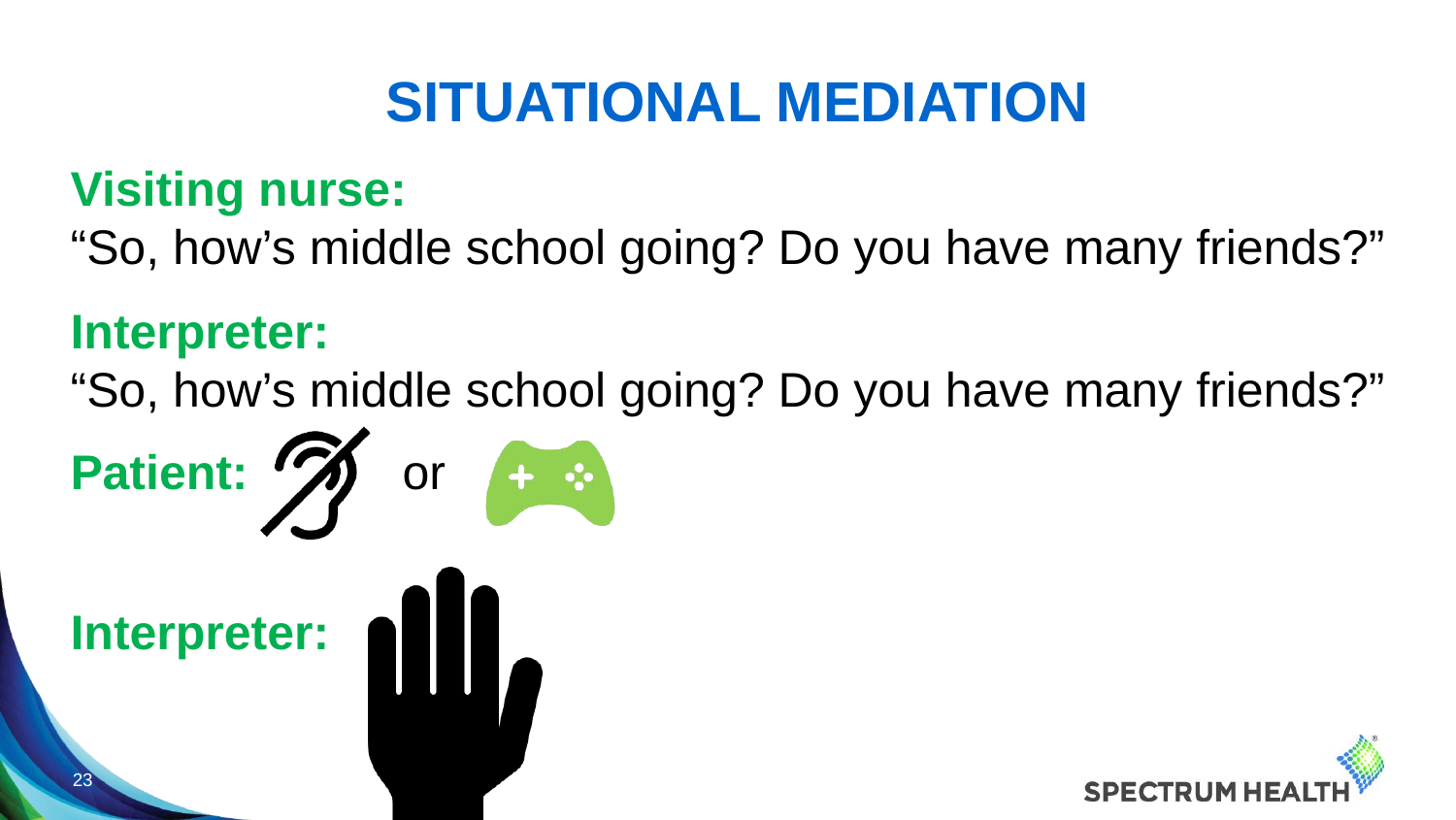

SITUATIONAL MEDIATION
Visiting nurse:
“So, how’s middle school going? Do you have many friends?”
Interpreter:
“So, how’s middle school going? Do you have many friends?”
Patient:	 or
Interpreter:
23

## Slide 24
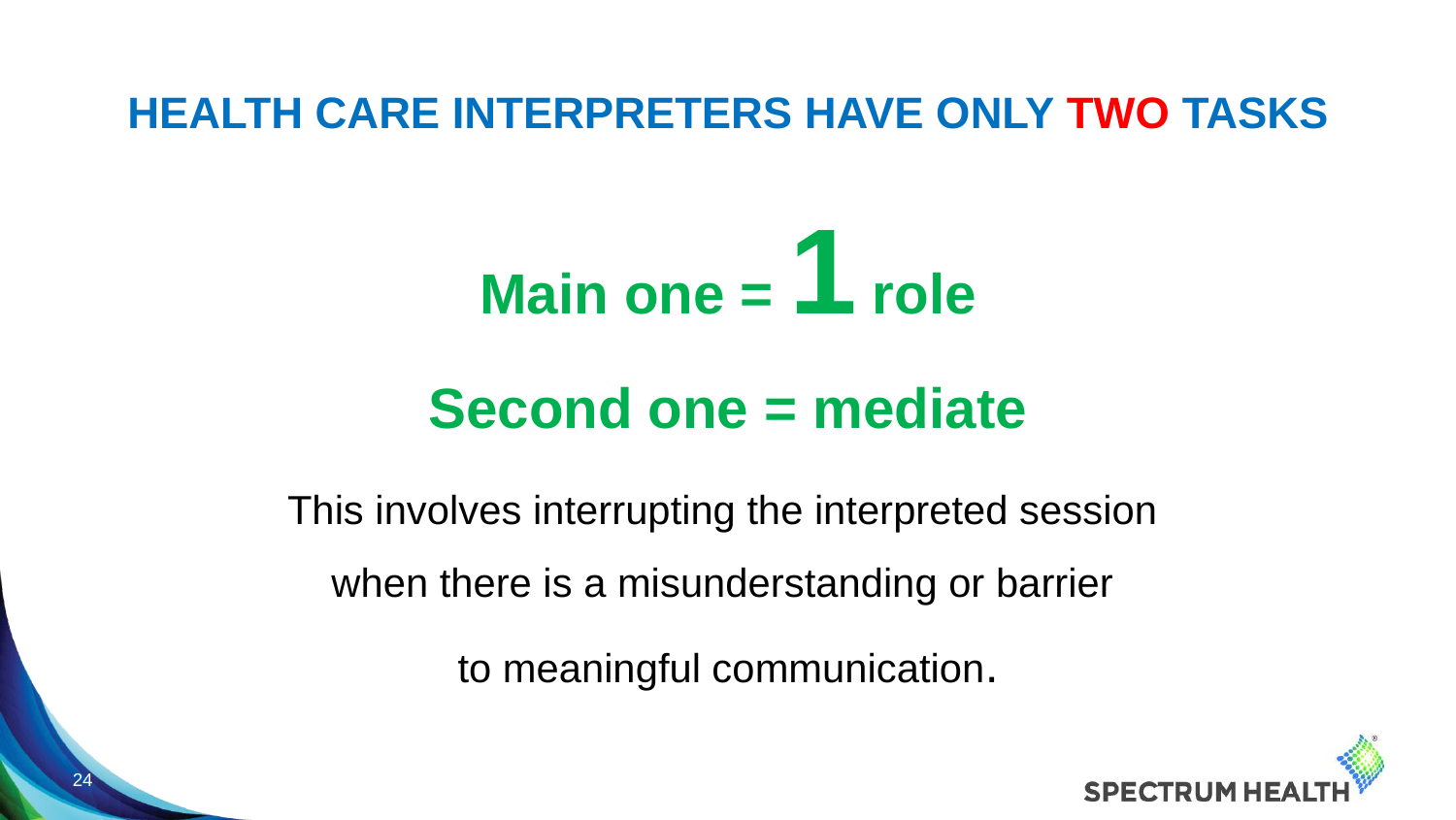

# HEALTH CARE INTERPRETERS HAVE ONLY TWO TASKS
Main one = 1 role
Second one = mediate
This involves interrupting the interpreted session
when there is a misunderstanding or barrier
to meaningful communication.
24

## Slide 25
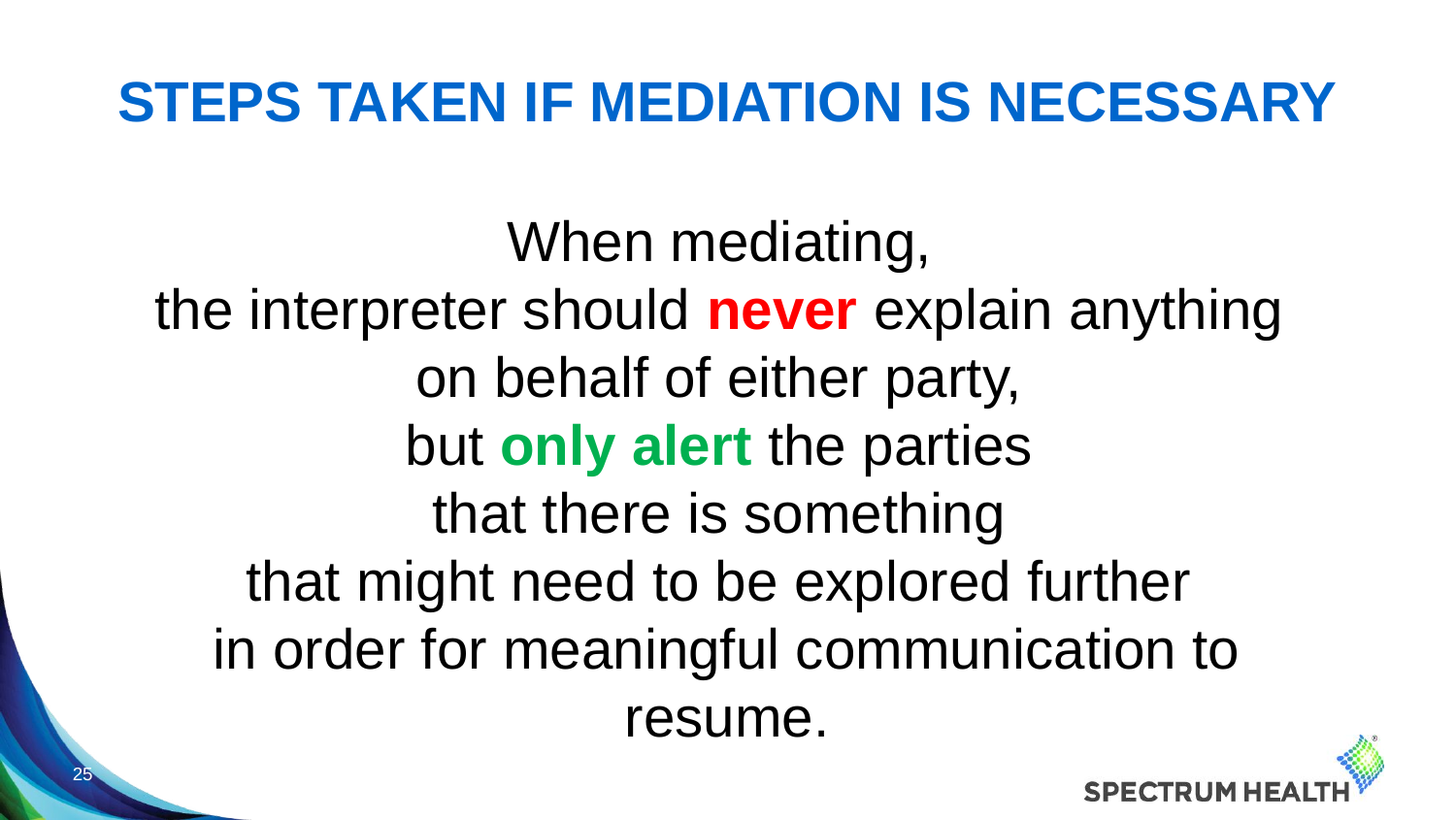

# STEPS TAKEN IF MEDIATION IS NECESSARY
When mediating,
the interpreter should never explain anything
on behalf of either party,
but only alert the parties
that there is something
that might need to be explored further
in order for meaningful communication to resume.
25

## Slide 26
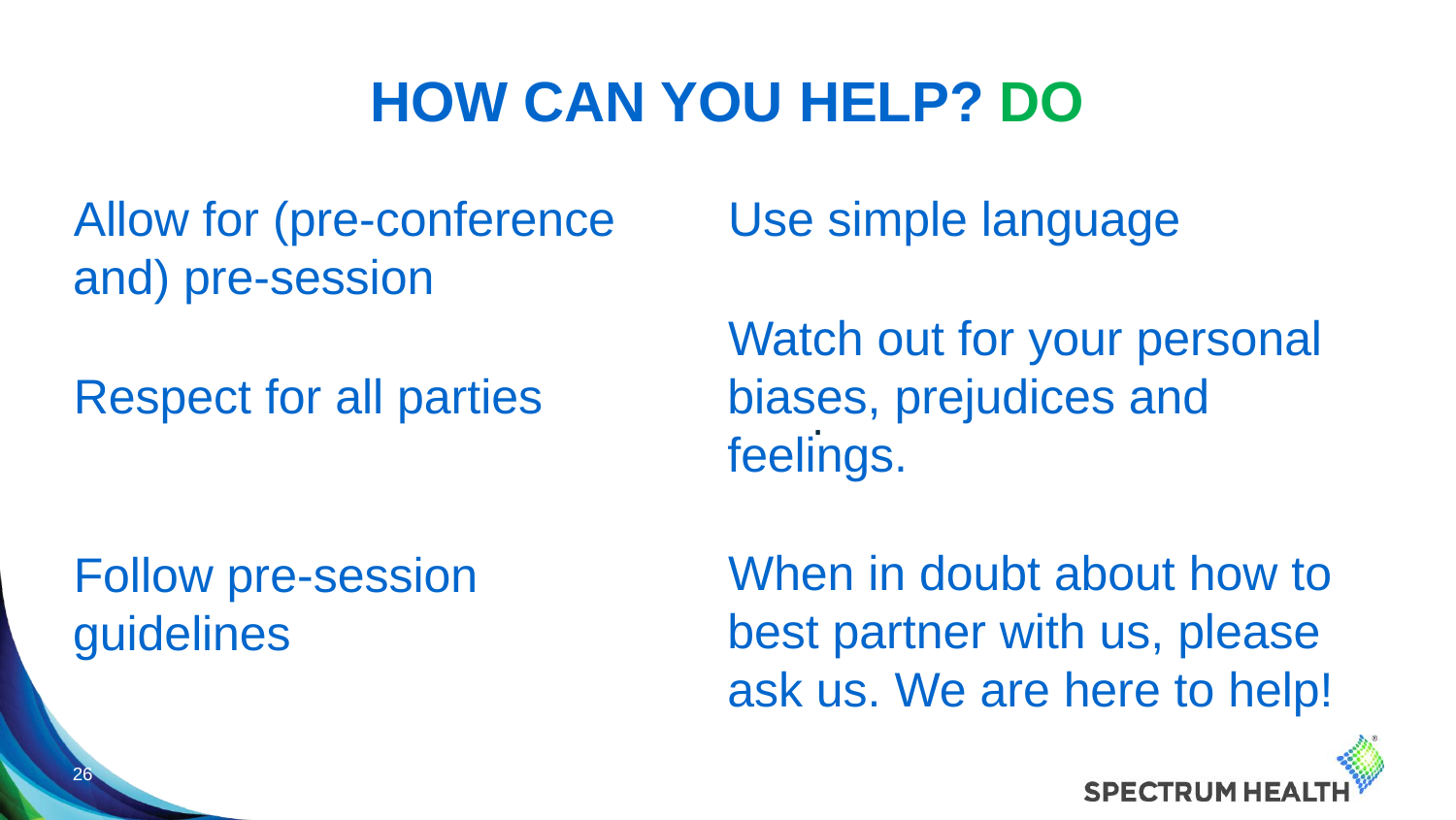

# HOW CAN YOU HELP? DO
Allow for (pre-conference and) pre-session
Respect for all parties
Follow pre-session guidelines
Use simple language
Watch out for your personal biases, prejudices and feelings.
When in doubt about how to best partner with us, please ask us. We are here to help!
.
26

## Slide 27
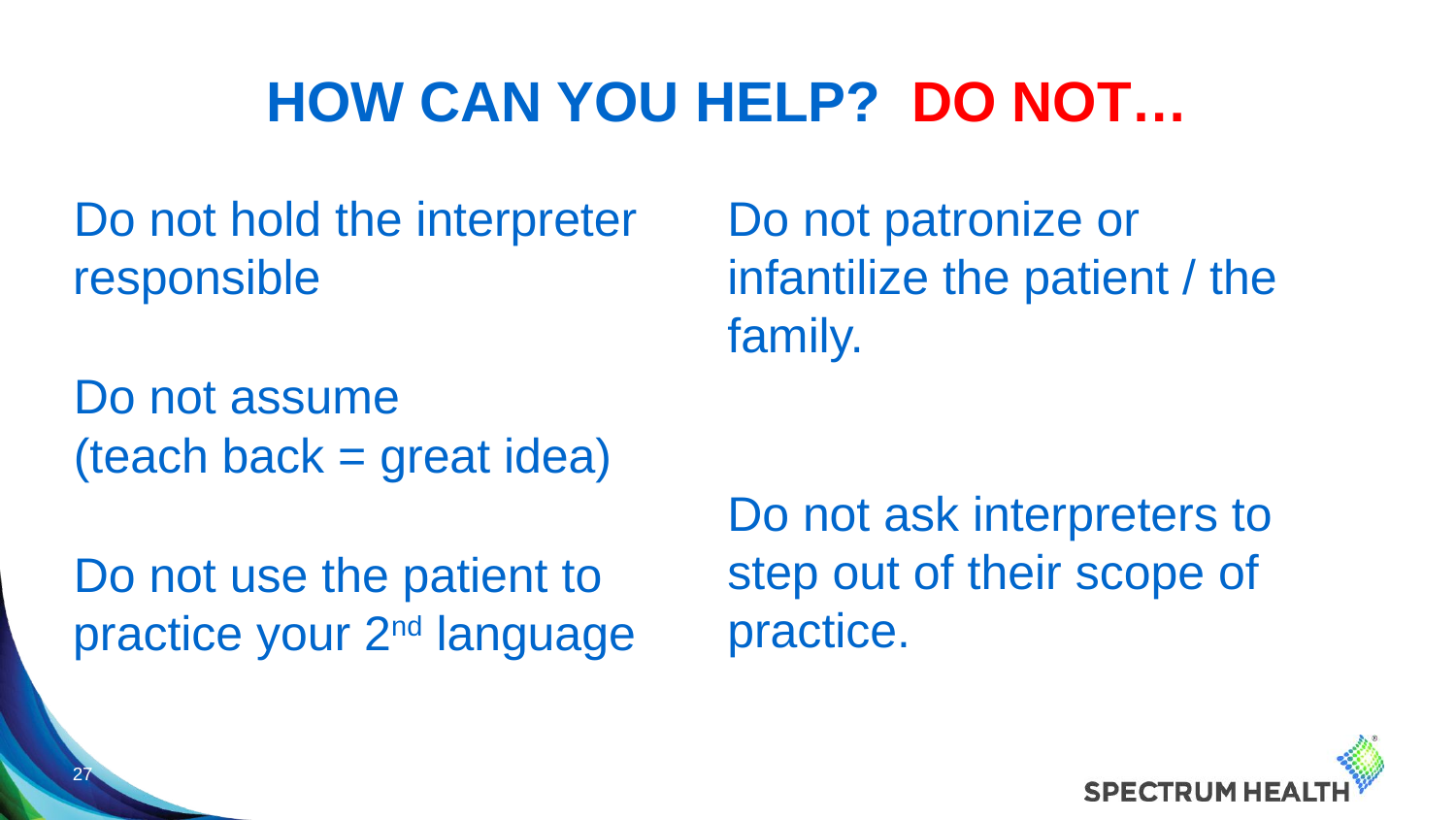

# HOW CAN YOU HELP? DO NOT…
Do not hold the interpreter responsible
Do not assume
(teach back = great idea)
Do not use the patient to practice your 2nd language
Do not patronize or infantilize the patient / the family.
Do not ask interpreters to step out of their scope of practice.
27

## Slide 28
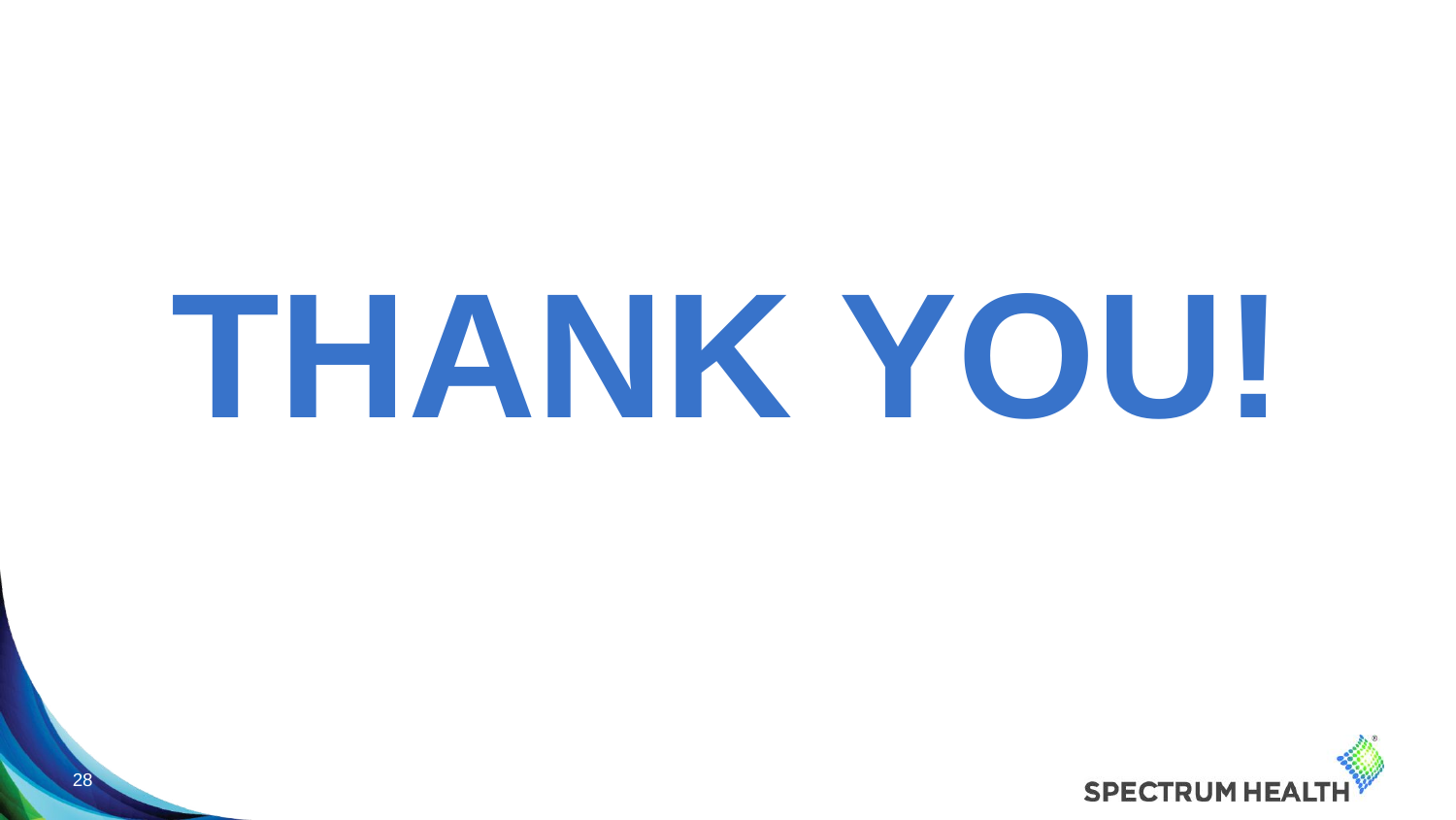

THANK YOU!
28

## Slide 29
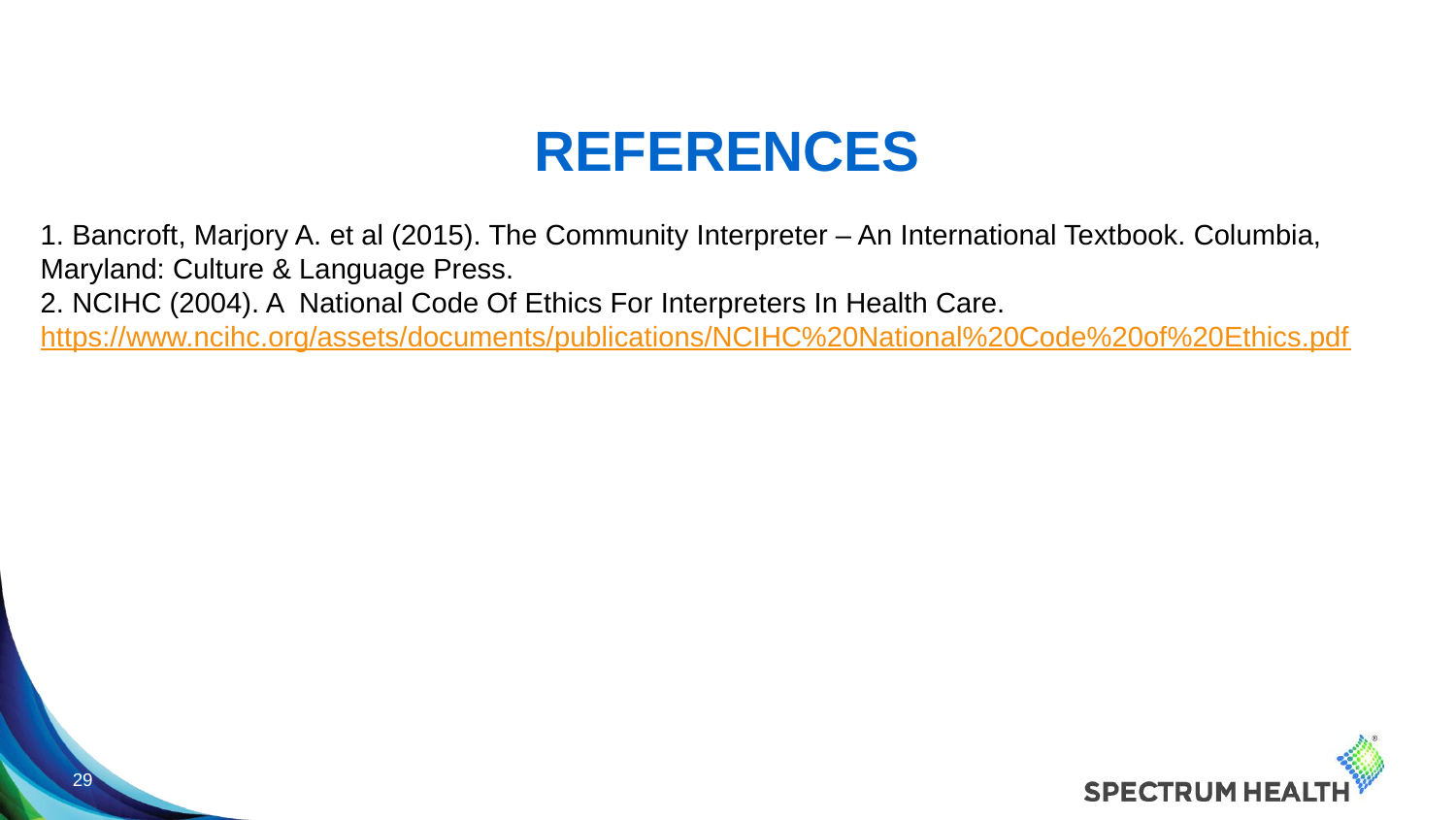

REFERENCES
1. Bancroft, Marjory A. et al (2015). The Community Interpreter – An International Textbook. Columbia, Maryland: Culture & Language Press.
2. NCIHC (2004). A National Code Of Ethics For Interpreters In Health Care.
https://www.ncihc.org/assets/documents/publications/NCIHC%20National%20Code%20of%20Ethics.pdf
29
