# Supplementary material for: Lost in Translation: An OSCE-Based Workshop for Helping Learners Navigate a Limited English Proficiency Patient Encounter
Source: MedEdPORTAL. 2021 Mar 17;17:11118. doi: 10.15766/mep_2374-8265.11118 (PMC7970641; doi:10.15766/mep_2374-8265.11118)
Supplement: Supplementary file 1 — Description of Workshop Components.docxChecklist.docxPreworkshop OSCE.docxPanel Discussion.docxWorking With Health Care Interpreters.pptxMap of Postworkshop OSCE.docxFacilitator Guide for Interactive Q&A.docxDebriefing.docxPostworkshop OSCE.docx [file mep_2374-8265.11118-s001.zip › F. Map of Postworkshop OSCE.docx]

**Appendix F:** Map of the Post-Workshop OSCE

| Time | Room 1 | Room 2 | Room 3 |
| --- | --- | --- | --- |
| Facilitators: | A & B | C & D | E & F |
| 2:30 – 2:40 | Learner 1 | Learner 5 | Learner 9 |
| 2:45 – 2:55 | Learner 2 | Learner 6 | Learner 10 |
| 3:00 – 3:15 | Learner 3 | Learner 7 | Learner 11 |
| 3:20- 3:30 | Learner 4 | Learner 8 | Learner 12 |
