# Supplementary material for: Lost in Translation: An OSCE-Based Workshop for Helping Learners Navigate a Limited English Proficiency Patient Encounter
Source: MedEdPORTAL. 2021 Mar 17;17:11118. doi: 10.15766/mep_2374-8265.11118 (PMC7970641; doi:10.15766/mep_2374-8265.11118)
Supplement: Supplementary file 1 — Description of Workshop Components.docxChecklist.docxPreworkshop OSCE.docxPanel Discussion.docxWorking With Health Care Interpreters.pptxMap of Postworkshop OSCE.docxFacilitator Guide for Interactive Q&A.docxDebriefing.docxPostworkshop OSCE.docx [file mep_2374-8265.11118-s001.zip › G. Facilitator Guide for Interactive Q&A.docx]

**Appendix G: Facilitator Guide for the Interactive Question and Answer Session**

Session begins with video titled, “Lost in Translation: Dos and Don'ts”
<https://mediaspace.msu.edu/media/t/1_qpuywdu3>

In this video, the following aspects were not done correctly by the physician:

- iPad interpreter not ideally positioned for physician and patient
- Does not ask questions one at a time
- Does not speak in “digestible chunks”
- Does not ask questions in first person (“Can you ask *her*…)
- Does not maintain eye contact with patient
- Uses medical terminology and uses jargon (e.g., hematochezia)

After the ~ 3-minute video ends, the session is intended to be interactive with open discussion. We include some sample questions with answers to help facilitate this portion.

**Asked by the facilitator:**

1. What went will in this encounter?
   - *Physician introduces self*
   - *Physician used an interpreter*
   - *Physician did a debrief with the interpreter (“Doc sent her in for her diabetes”)*
2. What could have gone better in this encounter?
   - *Physician was frowning (body language)*
   - *Physician interrupts the interpreter and patient*
   - *Make the patient more comfortable before examining her*
3. If the physician in the video was your trainee, what specific feedback would you give them?
   - *“I would….”*
4. How do you think the patient felt during this encounter? Why?
   - *Anxious because…, frustrated because…, uncomfortable because…*
5. How do you think the physician felt during this encounter? Why?
   - *The physician felt frustrated due to lack of training, lack of experience, technology issues…*
   - *The physician felt uncomfortable due to unfamiliarity with the patient’s language or culture…*

**Sample questions from the audience:***(You can use these questions to engage the audience if they do not ask these questions themselves. We also provide example answers, although some answers will vary on your institution.)*

1. How can we best partner with you?
   - *Introduce yourself to us and remember to debrief us prior to an encounter*
   - *Ask us for feedback and/or be open to receiving feedback*
   - *Remember that we are on the same team and want to provide the best care for the patient*
2. Do I need to use an interpreter even if I speak some (insert language here)?
   - *Yes, you should still use an interpreter if you are not proficient in the patient’s primary language*
   - *Some institutions can assess your level of proficiency and credential you to speak to the patient without using an interpreter*
3. Do I need to use an interpreter even if (insert language here) is my native language?
   - *This depends on your institution; some places will allow you to speak to the patient without an interpreter but will not allow you to act as an interpreter for another healthcare provider*
4. What’s the best way to get a hold of an interpreter?
   - *Our office number is…*
   - *We carry a pager…*
   - *We use this company…*
5. Can we schedule an interpreter for our outpatient visits?

- *Yes, please provide us with at least a 24 hour notice so that we can ensure an in-person interpreter can be present*

1. Can we schedule an interpreter for our inpatient family centered rounds?

- *Yes, please provide us with at least a 24 hour notice so that we can ensure an in-person interpreter can be present*

1. What should I do if a patient insists on not using an interpreter?

- *One technique is to state “I am sorry, but I do not feel like I would be providing you the best care unless I use an interpreter to explain…”*

1. What are ways I can build rapport with my patient even if we don’t speak the same language?

- *Use reassuring body language, maintain eye contact with the patient, and speak in an appropriate tone and volume…*
