# Supplementary material for: Lost in Translation: An OSCE-Based Workshop for Helping Learners Navigate a Limited English Proficiency Patient Encounter
Source: MedEdPORTAL. 2021 Mar 17;17:11118. doi: 10.15766/mep_2374-8265.11118 (PMC7970641; doi:10.15766/mep_2374-8265.11118)
Supplement: Supplementary file 1 — Description of Workshop Components.docxChecklist.docxPreworkshop OSCE.docxPanel Discussion.docxWorking With Health Care Interpreters.pptxMap of Postworkshop OSCE.docxFacilitator Guide for Interactive Q&A.docxDebriefing.docxPostworkshop OSCE.docx [file mep_2374-8265.11118-s001.zip › H. Debriefing.docx]

**Appendix G: Debriefing**

*Either the Facilitator(s) or the interpreters can lead this debriefing. This session takes place after the last scenario is performed and all the groups have reconvened. Please leave time to ask for open comments.*

1. Can you share one learning point that you took away from this workshop?
   1. Why was this a learning point for you?
   2. What made you say this?
2. Did the cases reflect real life scenarios that you have been a part of?
   1. What were the similarities and differences?
   2. In the real-life encounter, did you ask for an interpreter?
   3. If you had to do that real-life encounter again, what would you have done differently?
3. How will you change your current practice regarding LEP patients?
   1. Why is that an important change for you?
   2. Which aspect of the workshop made you aware of the changes that need to be made?
4. Any other feedback or comments?
   1. This feedback can be about the organization/flow of the workshop, about the interpreters, about the facilitators, what aspects they found more helpful, etc.
