# Supplementary material for: Lost in Translation: An OSCE-Based Workshop for Helping Learners Navigate a Limited English Proficiency Patient Encounter
Source: MedEdPORTAL. 2021 Mar 17;17:11118. doi: 10.15766/mep_2374-8265.11118 (PMC7970641; doi:10.15766/mep_2374-8265.11118)
Supplement: Supplementary file 1 — Description of Workshop Components.docxChecklist.docxPreworkshop OSCE.docxPanel Discussion.docxWorking With Health Care Interpreters.pptxMap of Postworkshop OSCE.docxFacilitator Guide for Interactive Q&A.docxDebriefing.docxPostworkshop OSCE.docx [file mep_2374-8265.11118-s001.zip › I. Postworkshop OSCE.docx]

| **Appendix I: Post-workshop OSCE**  **SIMULATION CASE TITLE: Making Students Aware of Language Services for Patient Encounter: Abscess**  **AUTHORS: Jan Fune, MD**  **LEARNER AUDIENCE: Pediatricians, pediatric residents, and medical students** | |
| --- | --- |
| **PATIENT NAME: David Atienza**  **PATIENT AGE: 12-year old**  **CHIEF COMPLAINT: Abscess**  **PHYSICAL SETTING: Emergency Department** | |
|  | |
| **Brief narrative description of case** | Patient is a 12-year old male who presents to the emergency department (ED) for a skin abscess. He was originally seen at an urgent care center and started on oral clindamycin but has not seen improvement. Today, it was tender to touch and redder, so his mom brought him to the ED for treatment. The attending ED physician notes that the abscess can likely be treated with a simple incision and drainage, and so he sends the learner in to obtain a history and physical, and to obtain consent for the procedure. The patient is English speaking, but Mom only speaks Spanish; Mom is accompanied by her friend.  The overall learner goals are to (1) identify the need for using an interpreter and (2) convey the importance of certified interpreters (versus using laypeople as ad hoc interpreters), and (3) work with the interpreter and caregivers in accordance to the guidelines presented in the workshop. |
| **Primary Learning Objectives** | - Recognize the need for language services - Explain need for language services to patient/caregiver(s) - Demonstrate consistent eye contact with patient/caregiver(s) - Exemplify speaking in digestible phrases |
| **Critical Actions** | The learner should:   - Introduce him/herself and explain his/her role - Identify the need for interpreter services within 90 seconds - Explain the reason for why an interpreter is needed - Place iPad/phone in appropriate location OR positions him/herself appropriately to work with the in-person interpreter - Briefly explain the purpose of the interview to the interpreter, especially for sensitive appointments - Ask the patient one question at a time - Present information at a pace that is easy to follow for both patient and interpreter; that is, give information in “digestible chunks” and/or allows appropriate pauses - Avoid using medical jargon and/or acronyms - Maintain direct eye contact with the patient instead of with the interpreter - Ask questions in the first person; e.g. “Do you feel…” - Utilize teach back to ensure patient comprehension - Lean on interpreter for cultural cues (e.g., patient does not want to be touched, no eye contact is normal, male speaking on behalf is normal, etc) - Nonverbal body communication should be reassuring; e.g., mannerisms, facial expressions, body language - Talks at an appropriate volume (e.g., does not talk louder due to working with an interpreter) - Address the issues that were of concern to the patient - Acknowledge and respond to the beliefs, concerns, and expectations about the patient’s problems |
| **Learner Preparation or Prework** | Patient is a 12-year old male presenting to the emergency department for a skin abscess. When you go to do the history and physical, the attending asks you to also obtain consent from his mom in case the abscess can be treated with a simple incision and drainage. |

| **Initial Presentation** | | | |
| --- | --- | --- | --- |
| **Initial vital signs** | BP 100/62, HR 90, RR 18, Temp 98.5F, Weight: 30 kg | | |
| **Overall Setting and Appearance** | The setting takes place in the emergency department. The patient is sitting in the bed holding onto his right arm as he does not want anyone to touch his wound. Mom is setting next to him and is conversing with another person in the room. | | |
| **Confederates (e.g., standardized participants) and their roles in the room at case start** | Patient’s mom and mom’s friend are present with the patient.  **Instructions for the parent**:   - Do not speak in English - Inform the learner that you do not understand why your son’s skin is not improving - Please appear anxious and ask several questions - Sample questions: What causes this? Why didn’t the antibiotics work? What is an incision and drainage? Will he be awake for this incision and drainage? - If the learner offers an interpreter, please insist that your friend can interpret for you - Only accept the interpreter once the learner has stated why a certified one is needed - If the resident uses medical jargon, tell them that you do not understand - Optional: may add cultural competency piece to encounter, such as concern for evil eye   **Instructions for the parent’s friend:**   - Do not introduce yourself unless the learner asks to identify you - Remember not to act as if you are a professional or certified interpreter - Interpret only segments of the dialogue - Do not do exact translations of mom’s dialogue - Add your own questions, comments, or dialogue apart from mom’s   **Instructions for patient:**   - Appear anxious - Start interpreting for learner and mom if learner does not identify need for an interpreter   **Instruction for interpreter:**   - Only enter the encounter if the learner requests an interpreter - Introduce self to learner and parent - Do not ask the learner for a description of the encounter or reason for interpreter request (learner should provide this information to you unprompted) - May interpret consecutively or simultaneously   **Instruction for observer:**   - Position yourself in such a way that you can clearly see all the participants’ faces in the room   **Parent’s opening statement**: “His wound keeps getting worse. I do not understand why.” | | |
| **HPI** | **Volunteered:**   - We went to an urgent care center 2 days ago and received clindamycin - Patient has been compliant with medications   **Must be asked:**   - Fever – no fever; last temperature taken was 98.6F at home (oral) - Patient’s past medical history – eczema - Patient’s activities? – Goes to school, plays football - Previous skin infections? – None - Has he ever had an incision and drainage done before? – No | | |
| **Past Medical/Surgical History** | **Medications** | **Allergies** | **Family History** |
| None | Clindamycin | None | Mom used to get skin infections but has not had any in over 1 year |
| **Physical Examination** | | | |
| **General** | Well appearing, adequately hydrated | | |
| **HEENT** | Normocephalic, extraocular movements intact, nares clear, throat unremarkable | | |
| **Neck** | Supple, no lymphadenopathy | | |
| **Lungs** | Clear to auscultation bilaterally | | |
| **Cardiovascular** | Regular rate and rhythm, normal S1/S2, no murmurs | | |
| **Abdomen** | Soft, nondistended, no organomegaly | | |
| **Neurological** | Normal tone, normal reflexes, normal strength | | |
| **Skin** | Right forearm has a 3 cm x 3 cm fluctuant skin lesion with mild erythema | | |
| **GU** | Normal male genitalia, testes descended bilaterally | | |
| **Psychiatric** | Appears anxious | | |

| **Instructor Notes - Changes and CASE Branch Points** | | |
| --- | --- | --- |
| **Intervention / Time point** | **Change in Case** | **Additional Information** |
| If learner does not explain the reason for why an interpreter is needed… | Mom should insist on using friend or friend can insist on interpreting | This is because we want the learners to be aware of why an interpreter is needed (e.g., for legal purposes, for communication efficiency) |
| If learner clearly states why an interpreter is needed… | A certified interpreter may enter the encounter | We had the faculty observer cue the interpreter to enter the room at this step |
| If need for interpreter is not identified within 90 seconds… | Behavior of the mom and/or friend gets strange to help learner realize mistake | Examples: appearing more anxious or getting upset |
| If learner does not identify each person in each room… | Do not offer your name/role to learner | Some of our residents assumed the mom’s friend was the interpreter or was the parent. In this case, do not have the mom’s friend clarify that she is not the parent. |
| If learner does not maintain direct eye contact with the patient/caregiver… | Interpreter may redirect him/her to keep eye contact with patient/caregiver | The interpreter can simply state: “Please direct your eye contact with the parent or patient” |
| If learner uses the patient as an interpreter… | The patient should appear more anxious and have a clearly difficult time translating medical words | For example, it is unlikely that a child would know what an abscess is, or know how to translate that word into another language |
| If the learner attempts to get consent without using an interpreter… | The parent should refuse to sign the consent form | If no interpreter is requested, only provide the English version of the consent form |

**Ideal Scenario Flow**

The learner enters the room and sees the patient sitting bed. The resident should introduce him/herself and explain his/her role. The learner should identify the need for a certified interpreter and should clearly explain to the patient, parent, and friend in the room why it is important. The learner should perform the critical actions (e.g., maintain eye contact with parent, avoid medical jargon, etc) once the interpreter joins the encounter. After addressing mom’s questions and concerns, the learner should use teach back to ensure comprehension. The encounter should end after the learner obtains consent from the parent using an interpreter.

**Anticipated Management Mistakes**

- The learner relies on mom’s friend to interpret: We found that some of the learners assumed that the mom’s friend was an interpreter already waiting for them in the room. If the learner did not ask who each person was in the room, they often failed to recognize who was the parent in the encounter. This was avoided by having mom hold the baby from the beginning of the encounter.
- The learner does not identify the need for the interpreter and uses the patient as an interpreter: We found that some learners assumed it was okay to use the patient to act as the interpreter between the patient, parent, and learner.
- Uncertainty of explaining an abscess: We found that some learners were hesitant to explain what causes an abscess if they were unsure of its pathophysiology. We also found that some learners thought that they were being graded on their medical knowledge, so we reminded them that this simulation focused on communication skills.
