# Supplementary material for: Lost in Translation: An OSCE-Based Workshop for Helping Learners Navigate a Limited English Proficiency Patient Encounter
Source: MedEdPORTAL. 2021 Mar 17;17:11118. doi: 10.15766/mep_2374-8265.11118 (PMC7970641; doi:10.15766/mep_2374-8265.11118)
Supplement: Supplementary file 1 — Description of Workshop Components.docxChecklist.docxPreworkshop OSCE.docxPanel Discussion.docxWorking With Health Care Interpreters.pptxMap of Postworkshop OSCE.docxFacilitator Guide for Interactive Q&A.docxDebriefing.docxPostworkshop OSCE.docx [file mep_2374-8265.11118-s001.zip › _Educational_Summary_Report_11118.pdf]

MedEdPORTAL® The AAMC Journal of Teaching and Learning Resources

Original Publication Open Access

Lost in Translation: An OSCE-Based Workshop for Helping Learners Navigate a Limited English Proficiency Patient Encounter

Jan Fune, MD*, Jennifer P. Chinchilla, Allison Hoppe, Chineze Mbanugo, Rachel Zuellig, Ali T. Abboud, Oselenonome Oboh, MS, J. M. Monica van de Ridder, MSc, PhD

*Corresponding author: Fune.jan@gmail.com

Abstract

Introduction: Residents have been known to report a lack of self-efficacy in their ability to provide care for limited English proficiency (LEP) patients. Interpreters must be utilized to help navigate these patient encounters, but many institutions do not have a curriculum focused on utilizing interpreters effectively. **Methods:** We created a 3-hour workshop for physician learners working with the pediatric population. It included a panel discussion, best-practices presentation, video demonstration, observing scenarios, and pre- and postworkshop objective structured clinical exams (OSCEs). The first OSCE introduced learners to a scenario (4-day-old with jaundice with an LEP parent) where interpreter use was imperative. The second OSCE allowed learners to perform another case (12-year-old with an abscess with an LEP parent) and practice newly obtained skills from the workshop. Both OSCEs were scored using a 16-item yes/no checklist. All pediatric residents filled out an eight-item survey to evaluate the workshop; a subset of that group performed the pre- and postworkshop OSCEs. **Results:** Forty pediatric residents attended the workshop and completed the survey. The workshop was well received, with the majority of residents stating they would change their current interpreter usage practices. Ten pediatric residents performed the pre- and postworkshop OSCEs; all improved their scores. **Discussion:** The workshop was effective in improving how residents navigated LEP encounters. It is applicable to learners of all levels who want to improve their communication skills to provide better care for LEP patients and can be tailored to fit the needs of a specific institution.

Keywords
Pediatrics, Interpreter, Spanish, Vietnamese, Limited English Proficiency, OSCE, Clinical Skills Assessment/OSCEs, Communication Skills, Cultural Competence, Diversity, Inclusion, Health Equity

Educational Objectives

By the end of this workshop, learners will be able to:

1. Compare the roles and responsibilities of translators and interpreters working in health care.
2. Evaluate clinical situations to determine if an interpreter is required for a patient encounter.
3. Describe techniques to improve limited English proficiency encounters (as described in the 16-item checklist).
4. Demonstrate best practices for partnering with an interpreter by performing at least 75% of the items on the checklist during the postworkshop objective structured clinical exam.

Citation:
Fune J, Chinchilla JP, Hoppe A, et al. Lost in translation: an OSCE-based workshop for helping learners navigate a limited English proficiency patient encounter. *MedEdPORTAL*. 2021;17:11118. https://doi.org/10.15766/mep_2374-8265.11118

Introduction

Between 1990 and 2013, the limited English proficiency (LEP) population grew 80% to nearly 25.1 million people in the United States.[1] Despite equal desires for clinical information, minority and lower income patients receive less information, less positive and reinforcing speech, and less talk overall from their health care providers.[2] The compromised ability to exchange information between physicians and LEP patients has been known to increase adverse events and medical errors during hospitalization and to poorly affect care coordination.[3,4] Furthermore, prior studies have shown that language barriers can also negatively impact residents by increasing their stress and the length of resident workdays, as well as affecting their ability to teach junior house staff and medical students.[5]

Copyright © 2021 Fune et al. This is an open-access publication distributed under the terms of the Creative Commons Attribution license.

The Accreditation Council for Graduate Medical Education (ACGME) and the American Board of Pediatrics (ABP) require residents to master communication with patients and families across a broad range of socioeconomic and cultural backgrounds.[6] However, many institutions do not have a standardized curriculum to master these communication skills, and some trainees may go through residency without opportunities to learn these crucial skills. The consequences of this deficiency in training are evidenced in studies in which physicians report low self-efficacy in providing care for LEP patients, a population already vulnerable to receiving poorer patient care experiences and worse health outcomes.[3,7,8] Interpreters should be considered part of the patient care team, but lack of physician training on optimally utilizing language services limits their efficacy and creates barriers beyond language.

The effective use of language is vital to the physician-patient relationship.[9] Thus, physicians need to adapt and become more proficient using language services for LEP patients. However, implementation of interpreter services alone does not always ensure effective use. Additionally, patient preferences and physician adoption of interpreter services still play a significant role in why these modalities may not be successful. New technologies, such as telephone services and video calling, offer further expansion of interpretation services, but their use is still underutilized, and there has been only limited training with them, particularly amongst residents and faculty.[10-12] A prior study created a curriculum on language interpretation within a medical school; its results showed increased proficiency in the use of telephone interpretation services.[11] We hoped to create a learning workshop that would increase residents' proficiency using both telephone and in-person interpreting services and reduce their perceived frustration during LEP encounters.

An open discussion with the pediatric residents at our institution revealed multiple deficiencies. Some did not know they were legally obligated to use an interpreter for LEP patients, some were not aware of all the services our interpreters provided (e.g., interpreting in person vs. phone, being able to schedule them for appointments), and some were not aware that a language service department existed within our institution. Importantly, we discovered that all of them wanted to improve their encounters with LEP patients. This information helped us develop our workshop and focus on creating a checklist that would help providers improve their interactions with the interpreter and patient.

Therefore, these three overarching themes inspired us to create the workshop: (1) the growing LEP population in the US, (2) reducing physician frustration when caring for LEP patients, and (3) meeting ACGME competency in communication skills.

Our institution, like many others, did not have a curriculum or standardized method of tracking residents on this previously mentioned ACGME milestone. We offered this workshop to help the program evaluate its residents and to help the residents achieve mastery of communication with LEP patients. The module includes a workshop comprising (a) a panel discussion, (b) a presentation on best practices, (c) watching a video demonstration, (d) observing scenarios, and (e) an interactive question session facilitated by the interpreters. Additionally, we created two objective structured clinical exams (OSCEs) on communication with LEP patients. The workshop aims to help residents understand the difference between interpreters and translators, recognize scenarios where use of interpreters was appropriate, and learn techniques to overcome language barriers by partnering with interpreters.

We reviewed existing *MedEdPORTAL* publications pertaining to physicians working with interpreters and sought to make our contribution to the existing literature. Prior publications focused on Spanish-speaking patients, and simulation cases had adult patients only.[13-15] Our module is unique in that it offers OSCE scenarios focusing on communication skills to discuss a plan (rather than making a diagnosis), introduces cultural competency components, and includes a scenario participant who attempts to interpret in place of a certified health interpreter. The two different OSCEs also allow evaluators to compare a baseline score and a postworkshop score to evaluate learners. In addition, our workshop is unique in that Vietnamese and Tigrinya (the language used in our video demonstration) certified health interpreters also contributed to its development and to the education of the learners.

Methods

Development

Our team consisted of one hospitalist, one pediatric resident, eight medical students, the manager of language services, three certified health interpreters, and one process improvement specialist. We coordinated with the pediatric residency program director to incorporate this workshop into the residents' weekly didactic sessions. Once approval had been received, we designed the workshop (Appendix A) with guidance from our certified health interpreters. The scenarios, based on common pediatric illnesses (jaundice and abscess), were written by a

Copyright © 2021 Fune et al. This is an open-access publication distributed under the terms of the Creative Commons Attribution license.

pediatrician. The three second-year medical students on our team compared the two cases and stated that they were equally comfortable performing either of them at their level of training. Certified health interpreters helped modify the cases to add a cultural barrier component.

Equipment/Environment for the OSCE
A high-fidelity baby mannequin was required; however, a substitute such as a doll or figure representing a patient would be enough to perform this simulation. Indeed, such a substitute might be preferred over a high-fidelity mannequin, which could distract from the case.

The rooms were set up like the schematic included in the Figure. This let the audience see all the personnel in the OSCE and allowed the observer to score the participant and evaluate the participant's performance.

Personnel
Personnel for the OSCE included the following:

- A standardized patient to play the role of the parent fluent in another language (e.g., Spanish or Vietnamese).
- An actor or standardized patient trained to play the role of the parent's friend who was fluent in the same language as the parent.
- A certified health interpreter to interpret for the parent and participant.
- A faculty observer to score participants on the checklist.

Other personnel for the workshop included the following:

- One or two people to be timekeeper and help facilitate groups moving from room to room.
- Up to five people from different professions (e.g., an interpreter, a physician, a social worker) for the panel discussion.
- A single certified health interpreter to facilitate the interactive question-and-answer session.

Implementation
Recruitment and training: The certified health interpreters were recruited from our institution's language services department. We chose to use Spanish and Vietnamese interpreters for our OSCEs because they were the two most common non-English languages spoken by patients within our health care system. Faculty observers were recruited from the pediatric hospitalist division and trained on scoring the learners based on our 16-item checklist (Appendix B) for approximately 20 minutes. We had two 60-minute practice runs with the medical students, interpreters, actors, and faculty observers. The workshop facilitator met with the certified health interpreters and actors to ensure proper workflow and understanding of the material. One month prior to the workshop, we recruited 10 pediatric residents to participate in the preworkshop OSCE (Appendix C) to obtain baseline data.

Preworkshop OSCE: Participants were given a prompt (Appendix C) and instructed that the patient was hemodynamically stable and well overall prior to entering the simulation room. We reminded the participants that the simulation focused on communication skills rather than on assessing their medical knowledge or management decisions. We allotted 10 minutes to complete the case and advised the participants that a warning would sound at the 8-minute mark. Observers were positioned in the room so as not to interfere with the dynamic between the parent and the participant (Figure).

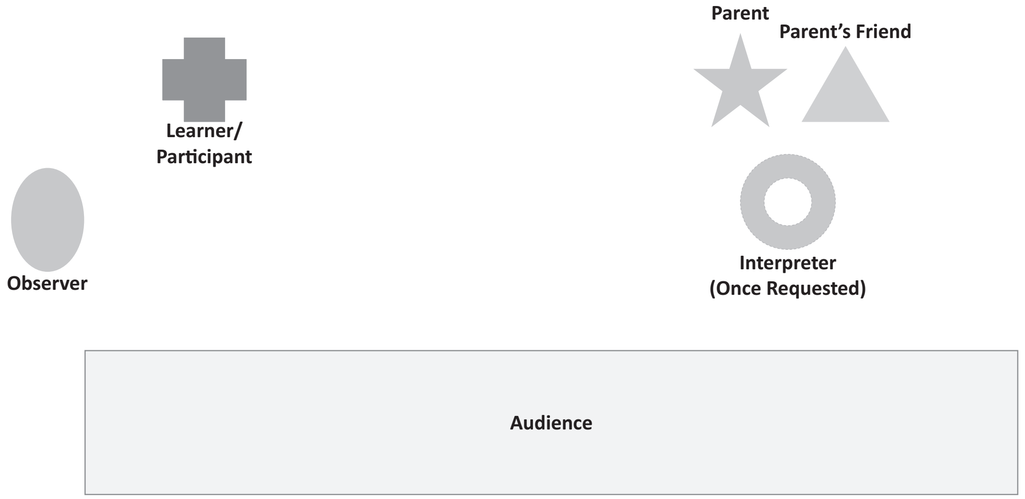

Figure. Recommended setup of objective structured clinical examination scenario.

Copyright © 2021 Fune et al. This is an open-access publication distributed under the terms of the Creative Commons Attribution license.

Each participant completed the preworkshop OSCE. Participants were not given feedback at the end of the case because we did not intend for this to be a learning intervention and were only obtaining baseline data. Therefore, the scores from the preworkshop OSCE were not shared with the participants to avoid focus on a failing or passing grade.

For both the pre- and postworkshop OSCEs, the case began as soon as a participant entered the room. To mimic real-life scenarios, the patient's friend started translating for the parent or the parent would rely on the friend to translate. If the participant did not recognize the need for a certified interpreter within 3 minutes, the parent or the parent's friend would give cues, such as appearing anxious or stating, "I do not understand what you are saying, doctor," or "I do not know how to translate that." Participants had to state why an interpreter was required for the encounter prior to the parent accepting the interpreter because, in some real-life scenarios, a physician may not think it is appropriate to use a certified interpreter. Once the interpreter entered the room, the parent became less anxious and answered questions appropriately, allowing the rest of the encounter to proceed more smoothly.

Workshop: The workshop was designed to be interactive and provide hands-on learning opportunities. We started with a panel discussion that included a Vietnamese interpreter, a Spanish interpreter, a social worker who primarily worked with the LEP population, and a pediatrician. The facilitator asked the panelists questions (Appendix D) and left time for other audience questions. This was followed by a PowerPoint presentation (Appendix E) led by a certified health interpreter to (1) define the difference between an interpreter and a translator; (2) explain the national code of ethics for interpreters in health care; (3) explain the reason why children, friends, and family should not facilitate LEP communication; and (4) describe the difference between simultaneous and consecutive interpreting. The presentation described how an ideal encounter with an LEP patient should be approached using an interpreter, which included items from our checklist (although the formal checklist seen in Appendix B was not presented). Afterward, the learners split into four groups so that the cases could be practiced in three separate rooms operating simultaneously (Appendix F), with the fourth room reserved for the interactive question-and-answer session with a certified health interpreter (Appendix G). To stimulate discussion, this group watched a video on an LEP encounter that went wrong due to improper utilization of a Tigrinyan interpreter. All groups rotated through the four rooms to ensure that all learners received the same content. The workshop ended with all four groups joining together for a 30-minute debrief about the cases, sharing their experiences and skills they had learned, and asking any other questions they might still have (Appendix H).

Postworkshop OSCE: The same 10 participants who completed our preworkshop OSCE were given the postworkshop OSCE to complete (Appendix I). This OSCE also focused on communication skills rather than medical knowledge and management and had a level of complexity similar to the preworkshop OSCE. Participants were told that this was the same setup as in the preworkshop OSCE but that they would be managing a different case. They were given the prompt before entering the patient's room (Appendix I). Participants were told that they had 10 minutes to complete the case and that a warning would sound at the 8-minute mark.

Immediately after completion of the postworkshop OSCE, 5 minutes were set aside for the interpreter to provide the participant with face-to-face feedback. Interpreters offered general comments on how effectively the participant had worked with the interpreter and communicated with the patient and parent. We encouraged the interpreters to use positive reinforcement to empower the participants and credit them for what they had performed successfully (e.g., "You did a good job of maintaining eye contact with the patient and parent"). Participants did not receive feedback on their diagnostic skills, medical management, or medical knowledge. Interpreters did not reveal a score to the participant; instead, they specified whether the participant had performed or missed items on our 16-item checklist (Appendix B). We deliberately did not share participants' scores as we did not want the score to be the motivation behind having an ideal encounter with LEP patients. The participants were also able to ask the interpreter and actor for specific feedback on their performance during this allotted time.

Assessment

Assessment of OSCE participants: The 16-item yes/no checklist (Appendix B) was created by our team of certified health interpreters, with input from the medical students on our team, and a pediatric hospitalist. The checklist was discussed until consensus was reached. A content expert in the area of training residents to work with LEP patients reviewed the checklist for content validation.[16]

We had three outcome measures for our OSCE participants: (a) All participants were scored on the checklist (range: 0 = minimum, 16 = maximum and a perfect score; all items were weighted equally); (b) faculty observers made specific notes

Copyright © 2021 Fune et al. This is an open-access publication distributed under the terms of the Creative Commons Attribution license.

on the checklist, which provided qualitative feedback on the participant's performance; and (c) faculty observers recorded the time it took for the participant to recognize that an interpreter was required for the encounter and request one.

Assessment of OSCEs: A medical student from our team interviewed postworkshop OSCE participants after they had received individual feedback from the interpreter. The purpose of this interview was to provide our team with feedback on how to improve the OSCE for future iterations of the workshop. Each interview took 5-10 minutes. Each participant was asked the following questions:

1. What were your thoughts on participating in the OSCE?
2. What did you learn from participating in the OSCE?
3. What worked or did not work during the OSCE?
4. How comfortable were you with the case you were given?
5. Other comments on the OSCE?

Results

Workshop Evaluation

The 40 residents who attended the workshop completed an eight-question survey at its end. Eighty-five percent ($n = 34$) graded the workshop as good or excellent, and 83% ($n = 33$) stated that they would change their current practice regarding LEP patients. The percentage of residents who rated their level of comfort in providing care to LEP patients as comfortable or very comfortable increased from 63% ($n = 25$) to 80% ($n = 32$). Open-ended questions were included in the survey. One stated, "As a result of what I learned from attending today's workshop, I intend to make the following practice/performance changes that I believe will result in more positive patient outcomes." Common responses included "use shorter sentences," "speak slower," "talk directly to the patient and parent," and "use a professional interpreter instead of a family member or friend." We also asked the attendees before and after the workshop if they considered interpreters part of the patient care team. Prior to the workshop, 28% ($n = 11$) somewhat agreed and 65% ($n = 26$) strongly agreed that interpreters were part of the patient care team. After the workshop, 7% ($n = 3$) somewhat agreed and 93% ($n = 37$) strongly agreed, reflecting a change in physician attitude toward interpreters.

Results of the Postworkshop OSCE Participants

We initially recruited 12 residents to participate in our preworkshop OSCE. Unfortunately, two were unable to attend the workshop, and so they did not perform the postworkshop OSCE. The 10 remaining participants who performed both OSCEs were pediatric residents: two PGY 3s and eight PGY 1s.

For the 10 pediatric residents who completed the pre- and postworkshop OSCEs, the checklist mean score increased from 10.1 to 13.8 ($p < .01$, two-sided paired t test). In addition, data from our checklist suggested that our workshop was successful due to the following aspects seen in the postworkshop OSCE:

- All participants referred to the interpreters by their correct title (instead of translator).
- All participants requested an interpreter instead of asking the friend to facilitate communication with the parent.
- The mean score increased to 13.8, with certain items such as maintaining direct eye contact with the patient/parent being performed by all participants.

The mean time to request an interpreter decreased from 207.0 to 58.5 seconds ($p < .01$, two-sided paired t test).

Discussion

Contribution to the Existing Literature

Although the ACGME and ABP require residents to master communication with patients and families across a broad range of socioeconomic and cultural backgrounds,[6] many institutions do not have a standardized curriculum for this. As a result, some trainees may go through residency without opportunities to learn these skills. With the growing LEP population, it is imperative that physicians receive training on the appropriate utilization of interpreters.[4] This workshop contributes to the existing literature by offering two OSCE cases that can be used for practicing how to work with an interpreter to overcome barriers to LEP patient care. The workshop also offers pre- and posttests to provide objective data on learner improvement using a peer-reviewed checklist. The checklist can additionally be used separately as a tool for teaching and as a physical tool for physicians to use during real encounters (e.g., the checklist can be placed near an interpreter phone as a visual reference). We believe this resource is a significant addition to the body of existing *MedEdPORTAL* publications that use simulations to teach similar skills.[13-15]

Reflections on Development and Implementation

This project was novel as it used a process improvement approach to promote proper utilization of interpreters and was developed by a team with diverse membership: a process improvement specialist, medical students, and interpreters. It was important to have the input of all the specialties represented in our team to make sure that we approached this problem from all perspectives and ultimately improved the dynamic between medical providers, interpreters, and patients. The process improvement specialist helped us stay accountable for the progression of the project by implementing a structured time

Copyright © 2021 Fune et al. This is an open-access publication distributed under the terms of the Creative Commons Attribution license.

line and deadlines. The process improvement specialist was also able to connect us with the proper stakeholders and, most notably, to get our language service providers involved from the very beginning of the project. Due to the nature of our process improvement department, we presented the progress of our project to a large audience every week and received feedback from other process improvement specialists, quality improvement specialists, and health care providers outside of our team (e.g., nurses, care managers). This ongoing feedback strengthened the development of the project. Our medical students contributed to the development of the intervention by participating in the weekly meetings and sharing their perspectives. One medical student described her experiences acting as an ad hoc interpreter for her parents at their medical visits and how these experiences altered the parent-child dynamic. Because of this, we emphasized the importance of not using children to interpret for their parents in medical situations during the presentation. The students were helpful in creating the physician survey, formulating the checklist, structuring the OSCEs, developing the cases, and completing a thorough literature search. The interpreters contributed their expertise to this project from conception to completion. They helped the team understand the role of an interpreter, their scope of practice, the training they had received to become certified health interpreters, and their code of ethics. They also helped us recognize aspects outside of language barriers, such as cultural factors, and showed us how they can help advocate for the patient. They were crucial in the development of workshop materials, providing critical feedback to the OSCEs, creating the checklist, and giving feedback to the learners who participated in the postworkshop OSCE.

The support and commitment we received from the residents and the pediatric residency program were vital. The information from our open discussion with the residents helped us to assess their knowledge deficit and desires to improve their communication skills. Developing and implementing this workshop required a significant time commitment, but the investment was worthwhile. The practice sessions with the interpreters and actors were essential to ensure that the checklist we created was applicable to the scenarios and could be completed in the allotted 10 minutes. The practice sessions also helped us to streamline the workflow and avoid confusion and errors.

Evaluation of Work and Reflection of Results

The checklist score was an objective way to track the progress of the OSCE participants. The 16-item checklist was reviewed by an expert in this field who agreed with the items we included. The preworkshop OSCE confirmed that residents were not navigating LEP patient encounters correctly. The preworkshop data showed several behaviors that suggested improper use: for example, not requesting an interpreter despite the patient speaking another language and using the patient's friend as an interpreter instead of using a certified interpreter. Two residents who were not native Spanish speakers attempted to obtain history without using an interpreter and spoke in broken Spanish prior to requesting one. Seven of the residents attempted to use the actor portraying the friend as an interpreter despite the actor being instructed to give poor interpretations. Furthermore, it was noticed that the term *translator* was consistently used incorrectly (e.g., "I need a translator to speak to you"). Feedback from the postworkshop OSCE participants ($n = 10$) revealed that the OSCE scenarios reflected real-life situations well and that the participants appreciated receiving feedback from the interpreter and having time to reflect upon the process afterward. Participants also suggested (1) including a chair for the interpreter in the room so that everyone would be at the same eye level, (2) providing more time for the encounters, and (3) including more cultural topics as part of the workshop.

Limitations

We identified several limitations in our study. We focused only on Spanish and Vietnamese, since they were the top two non-English languages spoken by patients in our health care system. Thus, the cultural competency components of the workshop were limited to these cultures; however, this can easily be modified based on the demographics of a particular hospital system. A second limitation was that learners experienced a different difficult cultural situation depending on which patient they received. However, the scenarios we provide could be adapted to fit learners' needs regardless of what language is focused on. Although the workshop taught skills on how to use different interpreter modalities, we only practiced in-person encounters and not iPad or phone use. Another limitation was the total duration of the workshop, which was only 3 hours. Many attendees gave feedback that they would have liked more hands-on experience, so an extra hour of workshop might have been beneficial. Also, since switching stations required a lot of coordination, it would have been better to have two facilitators instead of one to help keep track of time and places. Future iterations of the workshop would benefit from quicker transition times between stations and more dedicated time for practicing newly learned skills and tools. Finally, the fact that all 10 participating residents in the OSCEs were volunteers may have influenced the checklist score as participants may have been self-motivated to learn about providing care for LEP patients. They were also exposed to two learning situations, while

Copyright © 2021 Fune et al. This is an open-access publication distributed under the terms of the Creative Commons Attribution license.

the other 30 residents did not get to actively participate in the pre- or postworkshop OSCE.

Implications for Interpreters, Medical Students, Residents, and Residency Programs

While developing our project, we discovered that there were many assumptions the interpreters made about the residents and physicians. For instance, our interpreters were not aware that residents are already physicians but are still in training. The interpreters were also unaware that most physicians do not receive formal training on working with interpreters at either the medical school level or the resident education level. This led to multiple misunderstandings when designing the cases and workshop. Clarifying these misunderstandings gave the interpreters more insight into the role of residents as physicians. Having the certified health interpreters meet the residents face to face during the workshop and the interactive question-and-answer session helped foster a stronger relationship. It allowed for open communication between the interpreters and residents, giving them a safe space for open dialogue, which should result in a better dynamic between both professions. It also showed the interpreters that residents were open to their feedback, encouraging them to provide feedback in real-life encounters as well.

The project taught the students how to perform a robust process improvement project and to use the plan, do, study, act method to define a problem and work toward a solution. These students are likely to have an advantage compared to their peers in residency because they learned this skill earlier in their training and received a foundation in process improvement. Another significant implication was that the students were confronted by how much language services are underutilized when caring for LEP patients, as well as being exposed to some physicians' frustration regarding language barriers at an early stage in their medical training. Students also learned about certified health interpreters and how to partner with them at an earlier stage of training. Increased awareness of the nuances required to work with interpreters will hopefully affect how they care for LEP patients in the future. Several students were able to present this project at local conferences, reaching a wider audience outside of our institution, and had an opportunity to practice their presentation skills. Additionally, the students gained a deeper understanding of multidisciplinary projects. This skill is critical as medicine moves towards a more team-based approach.

The workshop made residents aware of the legal implications of not using an interpreter to communicate with their patients and the high risk of errors due to language discrepancies. Residents learned that interpreters do not translate word for word but rather interpret. When residents were asked how they would change their current practice regarding LEP patients, common themes they reported included taking cultural qualities into consideration when providing care, using a certified health interpreter even if a family member or friend offers to translate, maintaining eye contact and talking directly to the patient/parent, and speaking in digestible phrases. Specific to our institution, participants learned all the ways they could utilize our language service department, such as scheduling in-person interpreters to be present for family-centered rounds.

Future Directions

We are currently in talks with hospital leadership to provide this workshop to other residency programs in our health care system. Within the pediatric residency leadership, we are working to make the workshop part of the advocacy curriculum or onboarding experience for new interns. We hope to standardize the workshop as a mandatory part of onboarding for all patient-facing employees. We believe the workshop is applicable to a broader range of learner participants, such as preclinical and clinical medical students, nursing students, physician assistant students, as well as residents in other specialties and practicing physicians. The specifics of the case can be modified to fit the audience, but emphasis should remain on improving partnership with interpreters and practicing communication skills.

Second, as more departments offer our workshop, we hope that they become more aware of the extra time needed to have a proper physician-patient encounter using an interpreter. If clinics could increase their appointment visit time slots for LEP patients, it might help reduce both physician and patient frustration since time limitations can exacerbate language barriers and miscommunication.

Lastly, we believe that all health care providers want to provide the best care possible by seeking improvement in their conversational and interactional skills. Breaking language barriers and building rapport with patients and families are an important part of providing care. Requesting an interpreter earlier in a patient encounter and optimizing the interaction between physicians and interpreters have significant implications in the care and health outcomes of LEP patients. This workshop, which requires time and stakeholder investment, has the potential to significantly affect the quality of care provided to LEP patients and to improve the relationship between interpreters and health care providers.

Copyright © 2021 Fune et al. This is an open-access publication distributed under the terms of the Creative Commons Attribution license.

Appendices

A. Description of Workshop Components.docx
B. Checklist.docx
C. Preworkshop OSCE.docx
D. Panel Discussion.docx
E. Working With Health Care Interpreters.pptx
F. Map of Postworkshop OSCE.docx
G. Facilitator Guide for Interactive Q&A.docx
H. Debriefing.docx
I. Postworkshop OSCE.docx

All appendices are peer reviewed as integral parts of the Original Publication.

Jan Fune, MD: Second-Year Pediatric Hospital Medicine Fellow, Department of Pediatrics, Helen DeVos Children's Hospital; Clinical Instructor, Michigan State University College of Human Medicine; ORCID: https://orcid.org/0000-0002-0296-7309

Jennifer P. Chinchilla: Second-Year Medical Student, Michigan State University College of Human Medicine

Allison Hoppe: Second-Year Medical Student, Michigan State University College of Human Medicine

Chineze Mbanugo: Second-Year Medical Student, Michigan State University College of Human Medicine

Rachel Zuellig: Third-Year Medical Student, Michigan State University College of Human Medicine

Ali T. Abboud: Third-Year Medical Student, Michigan State University College of Human Medicine

Oselenonome Oboh, MS: Third-Year Medical Student, Michigan State University College of Human Medicine

J. M. Monica van de Ridder, MSc, PhD: Assistant Professor, Department of Emergency Medicine, Michigan State University College of Human Medicine; Development and Learning Specialist, Office of Research and Medical Education, Spectrum Health

Acknowledgments
Jennifer P. Chinchilla, Allison Hoppe, and Chineze Mbanugo contributed equally to this work and are co-second authors.

We would like to acknowledge our certified health interpreters, Edna Quartey, Daniela Iñiguez, CT, John Brown, and Miriam Andrade, for their expertise and training our residents; Terry Vander Lee, our process improvement specialist, who helped streamline this project and mentor us; Dr. Kira Sieplinga, the pediatric residency program director, for being invested in this and allowing us to incorporate this workshop as part of the residents' didactics; the diversity and inclusion residents at our institution, Drs. Amina Jaji, Tonia Afshan, and Nina Kuei, for leading an open discussion with the residents regarding language barriers, recruiting panelists, and their feedback on the workshop; Osose Oboh, Esaw Kurban, and Jennifer Jess, our other medical students, who contributed ideas and brainstormed with us in the planning phases of the project; Dr. Raquel Hernandez, our expert in communicating with patients with limited English proficiency; and Drs. Bhawana Arora, Julie Gunderson, Candace Smith-King, and Leora Mogilner, who helped us revise our manuscript.

Disclosures
None to report.

Funding/Support
None to report.

Prior Presentations
Perez C, Hoppe A, Mbanugo C, Fune J. Lost in translation. Presented at: Western Michigan University School of Medicine Health Equity Summit; August 17, 2019; Kalamazoo, MI.

Ethical Approval
Reported as not applicable.

References

1. Batalova J, Zong J. Language diversity and English proficiency in the United States. Migration Policy Institute. November 11, 2016. Accessed November 4, 2020. https://www.migrationpolicy.org/article/language-diversity-and-english-proficiency-united-states-2015
2. Hall JA, Roter DL, Katz NR. Meta-analysis of correlates of provider behavior in medical encounters. *Med Care*. 1988;26(7):657-675. https://doi.org/10.1097/00005650-198807000-00002
3. Quigley DD, Elliott MN, Hambarsoomian K, et al. Inpatient care experiences differ by preferred language within racial/ethnic groups. *Health Serv Res*. 2019;54(suppl 1):263-274. https://doi.org/10.1111/1475-6773.13105
4. Hartford EA, Anderson AP, Klein EJ, Caglar D, Carlin K, Lion KC. The use and impact of professional interpretation in a pediatric emergency department. *Acad Pediatr*. 2019;19(8):956-962. https://doi.org/10.1016/j.acap.2019.07.006
5. Chalabian J, Dunnington G. Impact of language barrier on quality of patient care, resident stress, and teaching. *Teach Learn Med*. 1997;9(2):84-90. https://doi.org/10.1080/10401339709539820
6. *The Pediatrics Milestone Project: A Joint Initiative of the Accreditation Council for Graduate Medical Education and the American Board of Pediatrics.* Accreditation Council for Graduate Medical Education; 2017. Accessed November 4, 2020. https://www.acgme.org/Portals/0/PDFs/Milestones/PediatricsMilestones.pdf?ver=2017-07-24-124802-340
7. Hernandez RG, Cowden JD, Moon M, Brands CK, Sisson SD, Thompson DA. Predictors of resident satisfaction in caring for

Copyright © 2021 Fune et al. This is an open-access publication distributed under the terms of the Creative Commons Attribution license.

limited English proficient families: a multisite study. *Acad Pediatr*. 2014;14(2):173-180. https://doi.org/10.1016/j.acap.2013.12.002

8. Eneriz-Wiemer M, Sanders LM, Barr DA, Mendoza FS. Parental limited English proficiency and health outcomes for children with special health care needs: a systematic review. *Acad Pediatr*. 2014;14(2):128-136. https://doi.org/10.1016/j.acap.2013.10.003
9. Woloshin S, Bickell NA, Schwartz LM, Gany F, Welch HG. Language barriers in medicine in the United States. *JAMA*. 1995;273(9):724-728. https://doi.org/10.1001/jama.1995.03520330054037
10. Lee JS, Nápoles A, Mutha S, et al. Hospital discharge preparedness for patients with limited English proficiency: a mixed methods study of bedside interpreter-phones. *Patient Educ Couns*. 2018;101(1):25-32. https://doi.org/10.1016/j.pec.2017.07.026
11. Omoruyi EA, Dunkle J, Dendy C, McHugh E, Barratt MS. Cross talk: evaluation of a curriculum to teach medical students how to use telephone interpreter services. *Acad Pediatr*. 2018;18(2):214-219. https://doi.org/10.1016/j.acap.2017.11.010
12. Diamond LC, Schenker Y, Curry L, Bradley EH, Fernandez A. Getting by: underuse of interpreters by resident physicians. *J Gen Intern Med*. 2009;24(2):256-262. https://doi.org/10.1007/s11606-008-0875-7
13. Callahan E, Garcia E, Rehm J. Talk louder? Communicating with your Spanish speaking patients. *MedEdPORTAL*. 2011;7:8427. https://doi.org/10.15766/mep_2374-8265.8427
14. Pinto Taylor E, Mulenos A, Chatterjee A, Talwalkar JS. Partnering with interpreter services: standardized patient cases to improve communication with limited English proficiency patients. *MedEdPORTAL*. 2019;15:10826. https://doi.org/10.15766/mep_2374-8265.10826
15. Zdradzinski MJ, Backster A, Heron S, White M, Laubscher D, Siegelman JN. A novel simulation to assess residents' utilization of a medical interpreter. *MedEdPORTAL*. 2019;15:10853. https://doi.org/10.15766/mep_2374-8265.10853
16. Downing SM. Validity: on the meaningful interpretation of assessment data. *Med Educ*. 2003;37(9):830-837. https://doi.org/10.1046/j.1365-2923.2003.01594.x

Received: June 10, 2020
Accepted: December 30, 2020
Published: March 17, 2021

Copyright © 2021 Fune et al. This is an open-access publication distributed under the terms of the Creative Commons Attribution license.
